# The prognostic importance of traumatic axonal injury on early MRI: the Trondheim TAI-MRI grading and quantitative models

**DOI:** 10.1007/s00330-024-10841-1

**Published:** 2024-06-19

**Authors:** Kent Gøran Moen, Anne-Mari Holte Flusund, Hans Kristian Moe, Nada Andelic, Toril Skandsen, Asta Håberg, Kjell Arne Kvistad, Øystein Olsen, Elin Hildrum Saksvoll, Sebastian Abel-Grüner, Audny Anke, Turid Follestad, Anne Vik

**Affiliations:** 1 Department of Circulation and Medical Imaging, Faculty of Medicine and Health Sciences, Norwegian University of Science and Technology (NTNU), 7491 Trondheim, Norway; 2 Department of Radiology, Vestre Viken Hospital Trust, Drammen Hospital, 3004 Drammen, Norway; 3 Department of Radiology and Nuclear Medicine, St. Olavs Hospital, Trondheim University Hospital, 7006 Trondheim, Norway; 4 Department of Neurosurgery, St. Olavs Hospital, Trondheim University Hospital, 7006 Trondheim, Norway; 5 Department of Neuromedicine and Movement Science, Faculty of Medicine and Health Sciences, Norwegian University of Science and Technology (NTNU), 7491 Trondheim, Norway; 6 Department of Radiology, Møre and Romsdal Hospital Trust, Molde Hospital, 6412 Molde, Norway; 7 Department of Neurosurgery, Oslo University Hospital, Rikshospitalet, P.O. Box 4950 Nydalen, 0424 Oslo, Norway; 8 Institute of Health and Society, Research Centre for Habilitation and Rehabilitation Models and Services (CHARM), Faculty of Medicine, University of Oslo, P.O. Box 1130 Blindern, 0318 Oslo, Norway; 9 Department of Physical Medicine and Rehabilitation, Oslo University Hospital, Ullevål Hospital, P.O. Box 4956 Nydalen, 0424 Oslo, Norway; 10 Department of Physical Medicine and Rehabilitation, St. Olavs Hospital, Trondheim University Hospital, 7006 Trondheim, Norway; 11 MI Lab and Department of Circulation and Medical Imaging, Norwegian University of Science and Technology (NTNU), 7491 Trondheim, Norway; 12 Department of Radiology, Nord-Trøndelag Hospital Trust, Levanger Hospital, 7600 Levanger, Norway; 13 Department of Rehabilitation, University Hospital of North Norway, 9038 Tromsø, Norway; 14 Faculty of Health Sciences, Department of Clinical Medicine, UiT- The Arctic University of Norway, 9038 Tromsø, Norway; 15 Department of Clinical and Molecular Medicine, Faculty of Medicine and Health Sciences, Norwegian University of Science and Technology, 7491 Trondheim, Norway; 16 Clinical Research Unit Central Norway, St. Olavs Hospital, Trondheim University Hospital, 7006 Trondheim, Norway

**Keywords:** Craniocerebral trauma, Trauma severity indices, Artificial intelligence, Diffuse axonal injury, Neuroimaging

## Abstract

**Objectives:**

We analysed magnetic resonance imaging (MRI) findings after traumatic brain injury (TBI) aiming to improve the grading of traumatic axonal injury (TAI) to better reflect the outcome.

**Methods:**

Four-hundred sixty-three patients (8–70 years) with mild (*n* = 158), moderate (*n* = 129), or severe (*n* = 176) TBI and early MRI were prospectively included. TAI presence, numbers, and volumes at predefined locations were registered on fluid-attenuated inversion recovery (FLAIR) and diffusion-weighted imaging, and presence and numbers on T2*GRE/SWI. Presence and volumes of contusions were registered on FLAIR. We assessed the outcome with the Glasgow Outcome Scale Extended. Multivariable logistic and elastic-net regression analyses were performed.

**Results:**

The presence of TAI differed between mild (6%), moderate (70%), and severe TBI (95%). In severe TBI, bilateral TAI in mesencephalon or thalami and bilateral TAI in pons predicted worse outcomes and were defined as the worst grades (4 and 5, respectively) in *the Trondheim TAI-MRI grading*. *The Trondheim TAI-MRI grading* performed better than the *standard* TAI grading in severe TBI (pseudo-*R*^2^ 0.19 vs. 0.16). In moderate-severe TBI, quantitative models including both FLAIR volume of TAI and contusions performed best (pseudo-*R*^2^ 0.19–0.21). In patients with mild TBI or Glasgow Coma Scale (GCS) score 13, models with the volume of contusions performed best (pseudo-*R*^2^ 0.25–0.26).

**Conclusions:**

We propose *the Trondheim TAI-MRI grading* (grades 1–5) with bilateral TAI in mesencephalon or thalami, and bilateral TAI in pons as the worst grades. The predictive value was highest for the quantitative models including FLAIR volume of TAI and contusions (GCS score <13) or FLAIR volume of contusions (GCS score ≥ 13), which emphasise artificial intelligence as a potentially important future tool.

**Clinical relevance statement:**

*The Trondheim TAI-MRI grading* reflects patient outcomes better in severe TBI than today’s standard TAI grading and can be implemented after external validation. The prognostic importance of volumetric models is promising for future use of artificial intelligence technologies.

**Key Points:**

- *Traumatic axonal injury (TAI) is an important injury type in all TBI severities. Studies demonstrating which MRI findings that can serve as future biomarkers are highly warranted.*
- *This study proposes the most optimal MRI models for predicting patient outcome at 6 months after TBI; one updated pragmatic model and a volumetric model.*
- *The Trondheim TAI-MRI grading, in severe TBI, reflects patient outcome better than today’s standard grading of TAI and the prognostic importance of volumetric models in all severities of TBI is promising for future use of AI.*

## Introduction

Traumatic axonal injury (TAI), or diffuse axonal injury, is a hallmark lesion type in traumatic brain injury (TBI). To diagnose TAI in clinical practice, early magnetic resonance imaging (MRI) is required [1, 2] Aberrant signals detected in predilection sites mostly in white matter (WM) on diffusion-weighted imaging (DWI) or fluid-attenuated inversion recovery (FLAIR), or microhaemorrhages on T2* gradient echo (T2*GRE) or susceptibility-weighted imaging (SWI), are all considered to serve as biomarkers of TAI [3]. TAI is a more serious finding when located deep in the brain, resulting from stronger forces impacting the brain. The progressive severity influences prognosis, and attempts have been made to grade TAI for clinical and research purposes. The *standard* TAI grading based on MRI is attributed to Gentry et al [4] and Adams et al [5] consists of three grades with increasing severity: Grade 1, TAI in hemispheres (including cerebellum); Grade 2, TAI in the corpus callosum; and Grade 3, TAI in the brainstem (including cerebellar peduncles). However, the prognostic value of this grading is not well established [6, 7]. Also, unilateral and bilateral TAI in the brainstem are graded equally, while recent studies have shown that bilateral brainstem lesions, in particular, are associated with poor outcomes [8, 9]. Further, TAI in the thalami and basal ganglia are closely associated with a worse outcome [10–12] but are not incorporated at all in the *standard* TAI grading.

In a related study, we demonstrated that bilateral TAI in the brainstem and thalami were associated with low Glasgow Coma Scale (GCS) scores [13]. Based on these findings, we proposed a TAI-MRI grading *reflecting injury severity* that, for the first time, included bilateral TAI in the brainstem or thalami as the worst grade. In the recently published Stockholm MRI grading, thalamic TAI as well as bilateral TAI in pons were incorporated, the latter defined as the worst grade [12].

In the present study, our main aim was to improve MRI grading of TAI, to better predict outcome. It should be noted that TBI patients may have TAI in different locations at the same time visible on different MRI sequences, making the statistical analyses challenging. We investigated the importance of location, number, and volumes of TAI on different early MRI sequences for prediction of outcome across all severities in TBI. Based on results from a set of statistical analyses, we aimed to develop a clinical Trondheim TAI-MRI grading and quantitative models. The prognostic performance of the new grading was compared to (1) the *standard* TAI grading [4], (2) the Stockholm MRI grading [12], (3) our recently proposed TAI-MRI grading *reflecting injury severity* [13], and (4) quantitative volumetric models. The study is part of the international TAI-MRI project (https://www.neuron-eranet.eu/projects/TAI-MRI/).

## Materials and methods

This study consisted of 463 patients (8–70 years) with TBI from three separate prospective cohorts (2004–2017, Fig. 1): (1) the Trondheim mild (m)TBI cohort [14], (2) the Trondheim moderate-severe (ms)TBI cohort [15], and (3) the Oslo severe TBI cohort [16]. We defined severe TBI by admission GCS scores ≤ 8, moderate TBI by scores of 9–13, and mTBI by scores of 14–15. We analysed the patients with msTBI jointly as well as splitting into groups with different injury severities. The worst computed tomography (CT) scans were scored according to the Marshall CT classification [13, 17]. See Supplemental Material.

**Fig. 1:**
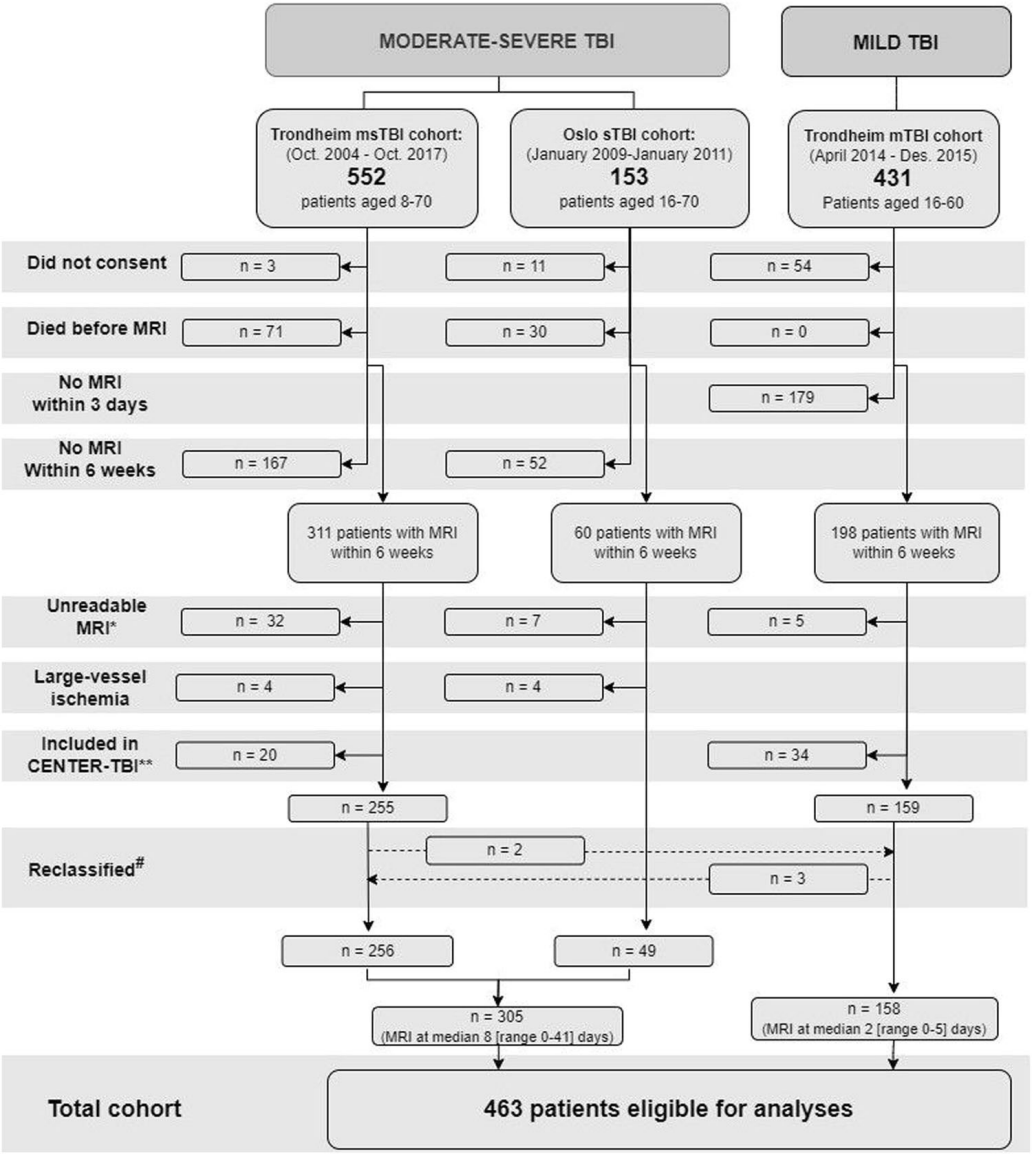
Flowchart of study inclusion and exclusion. TBI, traumatic brain injury; msTBI, moderate-severe TBI; sTBI, severe TBI; mTBI, mild TBI; Oct., October; Dec., December. *Due to poor quality or large artefacts, or missing one or more of three essential MRI sequences (fluid-attenuated inversion recovery, diffusion-weighted imaging, or T2* gradient echo/susceptibility-weighted imaging), since outcome analyses were performed with a complete case approach (see ‘Materials & methods’ section). **Excluded since CENTRE-TBI images will be used in later validation studies. ^#^Reclassified based on admission GCS score (see also ‘Materials & methods’ section)

### MRI acquisition, scoring, and annotation

Patients were scanned at 3 Tesla (*n* = 171), 1.5 Tesla (*n* = 287), or 1 Tesla (*n* = 5) within 6 weeks [18]. MRI scans were scored by consultants in radiology: The presence of TAI in 58 predefined locations including laterality was registered, and TAI lesions were counted on FLAIR, DWI (trace images), and T2*GRE (*n* = 266)/SWI (*n* = 204). If one or more of these three MRI sequences were missing, the patient was excluded (Fig. 1). Volumes of TAI on FLAIR and DWI were manually annotated using the 3D Slicer software package (version 4.8.0). For more details on how MRI findings including TAI lesions were detected, scored, and segmented, see Supplemental Material and Moe et al [13].

We defined contusions as either focal superficial lesions caused by the impact on brain parenchyma from dural/tentorial folds or bony structures [1] or, less common, uniform intra-axial haemorrhagic lesions measuring > 10 mm on FLAIR. Contusions were manually annotated and volumes on FLAIR were segmented with 3D Slicer [13].

The inter-rater agreements for the different cohorts have been reported earlier (positive and negative agreement for standard TAI grade ≥ 0.69 and intraclass correlation coefficients of different TAI numbers and volumes ≥ 0.78) [13, 14].

### Outcome assessments

Outcome was assessed with the Glasgow Outcome Scale Extended (GOSE) [19, 20] at 3 months in mTBI and 6 months in msTBI. In the Oslo severe TBI cohort, GOSE was administered at 3 and 12 months, and for these patients, the 6-month GOSE score was calculated as a weighted mean. Sixteen patients had missing GOSE scores, and we performed an imputation using the expectation-maximisation (EM) algorithm [21–23], see Supplemental Material.

In analyses of msTBI requiring a dichotomised outcome, GOSE scores were dichotomised into favourable (GOSE 5–8) or poor (GOSE score ≤ 4) outcomes. In separate analyses of patients with a GCS score of 13 and mTBI, GOSE scores were dichotomised into good recovery (GOSE score 7–8) or disability (GOSE score ≤ 6).

### Statistical analyses

#### Logistic regression analyses

In mTBI, the prognostic value of MRI variables was explored using *uni- and multivariable binary logistic regressions* with disability (GOSE score ≤ 6) as the response variable. Since few mTBI patients had a disability, each logistic regression analysis included only one MRI variable with age and sex as covariates. In msTBI, we investigated the prognostic value of MRI variables using *multivariable proportional odds ordinal logistic regressions* with the inverted GOSE score as the response variable. To comply with the IMPACT models, adjusted analyses in msTBI included the core variables (age, GCS score, and pupil dilation) and the Marshall CT score as covariates [24]. Since TAI on DWI may attenuate over time, we included an interaction term between DWI lesions and the number of days to MRI in one analysis. We also performed adjusted ordinal logistic regressions in severe TBI and moderate TBI with GCS scores of 9–12 separately and adjusted binary logistic regressions predicting disability (GOSE score ≤ 6) in moderate TBI with GCS score of 13 and mTBI. The results are presented as odds ratios (ORs) with 95% confidence intervals (CIs) and *p*-values. McFadden’s pseudo *R*^2^, the Akaike information criterion (AIC), and the Bayesian information criterion (BIC) were used to assess model fit.

#### Ordinal regression models with elastic-net penalty

Further, we studied msTBI in proportional odds ordinal regression models with elastic-net penalty, to investigate the *combined* prognostic effect of the TAI variables [25]. The type of penalty and the degree of shrinkage are controlled by two parameters that were selected by 5-fold cross-validation. The models were fitted by using the R package ordinalNet [26]. The uncertainty of the estimated coefficients was assessed by bootstrapping, showing the proportion of the 500 bootstrap samples where the variable was not shrunken to zero. We present two models: One with only TAI location (including laterality) variables *(elastic-net clinical TAI-MRI model)*, and one that also includes TAI number and volume variables *(elastic-net quantitative TAI-MRI model)*. Both models included core variables and Marshall CT score as covariates.

#### Comparison of the prognostic value of different TAI gradings in different TBI severities

Applying results from the different regressions described above, we propose the Trondheim TAI-MRI grading. The prognostic performance of four TAI gradings was compared using ordinal or binary logistic regression models that compromise core variables and Marshall CT score, and one of the following TAI gradings: (a) the *standard* TAI grading [4], (b) the Stockholm MRI grading [12], our recent (c) TAI-MRI grading based on GCS score [13], or (d) the Trondheim TAI-MRI grading. We also developed quantitative models comprising a volume of TAI and/or contusions which were compared with the models with TAI gradings (a–d). For the different regression models, the area under the receiver operating curve (AUC) for predicting poor outcome (GOSE score ≤ 4) in severe TBI and predicting disability (GOSE score ≤ 6) in moderate TBI, was calculated by 10-fold cross-validation [27], and presented with 95% bootstrap CI. Due to few MRI findings and few patients with disability, this analysis could not be performed in mTBI.

The statistical analyses were performed using IBM SPSS Statistics version 27, STATA/MP version 16.0, and R version 3.5.1 [28]. To give some protection against false positive results, a pragmatic approach was taken. Rather than using the commonly used limit of 0.05, *p*-values < 0.01 were regarded as statistically significant. Due to the explorative nature of the study, a formal adjustment with the risk of many false negatives was not desirable.

## Results

The presence of TAI differed between mild (6%), moderate (70%), and severe TBI (95%, Table 1). Bilateral TAI in the brainstem or thalami was only present in patients with severe TBI except for three patients with moderate TBI (Table 2). The total FLAIR volume of TAI was highest in severe TBI (median 1.29 cm^3^) and decreased significantly with lower injury severity (Table 1). Contusions were present in 4% of mTBI and 75% of msTBI.

**Table 1:** Demographics, injury and imaging variables, and outcome by TBI severity groups (*n* = 463)

Variable	Moderate-severe TBI (*n* = 305, 66%)			Mild TBI GCS score 14–15 (*n* = 158, 34%)
	Severe TBI GCS score 3–8 *(n* = 176, 38%)	Moderate TBI (*n* = 129, 28%)		
		GCS score 9-12 (*n* = 74, 16%)	GCS score 13^a^ (*n* = 55, 12%)	
Age (median, IQR)	27.2 (20.0–42.8)	32.5 (19.3–46.8)	31.1 (22.5–59.6)	27.7 (21.7–42.5)
Sex (male/female, %)	146/30 (83/17)	56/18 (76/24)	38/17 (69/31)	103/55 (65/35)
Injury mechanism (%)				
Road traffic accident	104 (59)	32 (43)	22 (40)	42 (27)
Fall	56 (32)	30 (41)	23 (42)	67 (42)
Struck object	4 (2)	0	1 (2)	17 (11)
Violence	8 (5)	2 (3)	5 (9)	22 (14)
Other/unknown	4 (2)	10 (14)	4 (7)	10 (6)
GCS score (median, IQR) (missing *n*)	6 (4–7) (1)	11 (10–12) (0)	13 (13–13) (0)	15 (15–15) (10)
Pupil dilatation at admission (*n*, %)				
Unilateral dilatation	37 (21)	1 (1)	1 (2)	0
Bilateral dilatation	9 (5)	1 (1)	0	0
Secondary events (*n*, %)				
Any hypoxia	64 (36)	7 (9)	1 (2)	0
Any hypotension	48 (27)	7 (9)	0	0
Alcohol intoxication at admission^b^ (*n*, %) (unknown *n*)	58 (33) (13)	26 (35) (0)	14 (25) (1)	72 (46) (2)
Worst Marshall CT score (*n*, %)				
No CT	0	0	0	31 (19)
1	11 (6)	11 (15)	13 (24)	148 (94)
2	77 (44)	49 (66)	25 (45)	10 (6)
3–4	43 (24)	5 (6)	4 (7)	0
5–6	45 (26)	9 (12)	13 (24)	0
Evacuated haematoma (*n*, %)	43 (24)	9 (12)	8 (15)	0
Days from injury to MRI (median, range)	9 (0–41)	7 (1–40)	6 (1–38)	2 (0–5)
TAI lesions on MRI (*n*, %)	167 (95)	55 (74)	35 (64)	9 (6)
Vol. TAI on FLAIR, cm^3^ (median, p75)	1.33 (5.36)	0.38 (1.76)	0 (0.58)	0 (0)
Vol. TAI on DWI, cm^3^ (median, p75)	0.43 (2.27)	0.10 (0.59)	0 (0.16)	0 (0)
No. TAI on T2*GRE/SWI (median, p75)	15 (39)	3.5 (21)	3 (21)	0 (0)
Contusions on MRI (*n*, %)	140 (80)	50 (68)	39 (71)	7(4)
Vol. Contusions, cm^3^ (median, p75)	5.11 (29.8)	3.02 (23.9)	5.26 (37.1)	0 (0)
Known preinjury disability^c^ (*n*, %, (missing *n)*)	31 (18) (3)	16 (22) (2)	4 (8) (1)	NA
Glasgow Outcome Scale Extended (GOSE) scores^d^ (*n*, %)				
1–2	15 (9)	0	0	0
3–4	44 (25)	5 (7)	1 (2)	0
5–6	79 (45)	30 (41)	19 (35)	16 (10)
7–8	28 (16)	34 (46)	34 (62)	131 (83)
Not possible to assess/missing	10 (6)	5 (7)	1 (2)	11 (7)

*TBI* traumatic brain injury, *n* numbers, *GCS* Glasgow Coma Scale, *IQR* interquartile range, *CT* computer tomography, *TAI* traumatic axonal injury, *Vol.* volume, *FLAIR* fluid-attenuated inversion recovery, *p75* 75th percentile, *DWI* diffusion-weighted imaging, *No*. number, T2*GRE T2* gradient echo, *SWI* susceptibility-weighted imaging

^a^ Patients with a GCS score of 13 were classified as moderate TBI

^b^ Positive blood alcohol content and/or clinical suspicion

^c^ Definition of preinjury disability: alcohol or/and drug abuse, psychiatric history, neurological disease (including epilepsy), developmental disorder or severe somatic disease (including cancer, severe heart- and lung disease)

^d^ 6 months GOSE score for the moderate/severe TBI cohort and 3 months GOSE score for the mild TBI cohort

**Table 2:** Adjusted^a^ ordinal logistic regression analyses with GOSE score^b^ as response variable and different TAI or contusion variables on MRI as explanatory variables

	All TBI			Severe TBI			Moderate TBI						Mild TBI^c^
	(*n* = 452)			GCS score ≤ 8 (*n* = 176)			GCS score 9–12 (*n* = 74)			GCS score 13 (*n* = 55)			GCS 14–15 (*n* = 147)
Location of TAI	*n* (%)	OR (95% CI)	*p-*value	*n* (%)	OR (95% CI)	*p-*value	*n* (%)	OR (95% CI)	*p*-value	*n* (%)	OR (95% CI)	*p-*value	*n* (%)
Bilateral TAI in brainstem/thalami	43 (10)	8.12 (4.32, 15.3)	**< 0.001**	40 (23)	5.58 (2.80, 11.2)	**< 0.001**	3 (4)	NA		0	NA		0
Bilateral TAI in pons	13 (3)	11.7 (4.07; 33.4)	**< 0.001**	12 (7)	10.7 (3.46; 33.2)	**< 0.001**	1 (1)	NA		0	NA		0
Bilateral TAI in mesencephalon/thalami	30 (7)	5.01 (2.49; 10.1)	**< 0.001**	28 (16)	3.82 (1.77; 8.27)	**0.001**	2 (3)	NA		0	NA		0
Any TAI in brainstem/thalami/basal ganglia^d^	128 (28)	3.82 (2.49; 5.87)	**< 0.001**	94 (53)	3.14 (1.78; 5.53)	**< 0.001**	22 (30)	2.91 (1.03; 8.28)	0.045	12 (22)	0.65 (0.14; 3.16)	0.59	0
Bilateral TAI in brainstem^d^	32 (7)	10.3 (4.96; 21.2)	**< 0.001**	29 (16)	6.01 (2.71; 13.3)	**< 0.001**	3 (4)	NA		0	NA		0
Unilateral TAI in brainstem	52 (12)	2.53 (1.44; 4.41)	**0.001**	40 (23)	1.30 (0.67; 2.54)	0.44	10 (14)	2.11 (0.50; 8.93)	0.31	2 (4)	NA		0
Bilateral TAI in thalami^d^	19 (4)	10.2 (4.18; 24.5)	**< 0.001**	19 (11)	8.24 (3.17; 21.4)	**< 0.001**	0	NA		0	NA		0
Unilateral TAI in thalamus	42 (9)	3.25 (1.73; 6.13)	**< 0.001**	31 (15)	3.09 (1.44; 6.62)	**0.005**	6 (8)	NA		5 (9)	NA		0
Bilateral TAI in basal ganglia^d^	16 (4)	7.93 (3.87; 20.6)	**< 0.001**	15 (9)	5.42 (1.95; 15.0)	**0.001**	1	NA		0	NA		0
Unilateral TAI in basal ganglia	43 (10)	1.82 (0.98; 3.37)	0.058	26 (15)	2.72 (1.22; 6.06)	0.015	8 (11)	NA		9 (16)	NA		0
TAI in corpus callosum^d^	143 (32)	2.77 (1.82; 4.23)	**< 0.001**	107 (61)	2.45 (1.38; 4.34)	**0.002**	21 (28)	0.78 (0.27; 2.24)	0.65	14 (25)	0.61 (0.13; 2.90)	0.53	1 (1)
TAI in hemispheres	252 (56)	0.85 (0.52; 1.41)	0.53	155 (88)	0.63 (0.26; 1.51)	0.30	54 (73)	0.88 (0.34; 2.32)	0.81	34 (62)	1.14 (0.31; 4.17)	0.84	9 (6)
**TAI volumes or numbers**													
Vol. TAI on FLAIR	227 (50)	1.78 (1.51; 2.10)	**< 0.001**	146 (83)	2.04 (1.65; 2.52)	**< 0.001**	50 (68)	1.42 (0.95; 2.11)	0.089	25 (45)	0.72 (0.31; 1.68)	0.45	6 (4)
Vol. TAI on DWI	176 (39)	1.87 (1.52; 2.30)	**< 0.001**	112 (64)	2.17 (1.66; 2.86)	**< 0.001**	40 (54)	1.14 (0.63; 2.04)	0.67	20 (36)	0.95 (0.46; 1.97)	0.89	4 (3)
No. TAI on T2*GRE/SWI	240 (53)	1.01 (1.01; 1.02)	**< 0.001**	149 (85)	1.01 (1.00; 1.02)	**0.001**	49 (66)	1.00 (0.98; 1.02)	0.86	33 (60)	0.99 (0.96; 1.02)	0.43	9 (6)
**Contusions**													
Contusions on CT	152 (34)	4.56 (3.16; 6.59)	**< 0.001**	90 (51)	1.43 (0.84; 2.44)	0.18	27 (36)	2.19 (0.88; 5.46)	0.092	30 (55)	9.13 (2.19; 38.0)	**0.002**	5 (3)
Contusions on MRI	236 (52)	7.42 (5.09; 10.8)	**< 0.001**	140 (80)	2.37 (1.24; 4.54)	**0.009**	50 (68)	1.66 (0.63; 4.36)	0.30	39 (71)	3.02 (0.53; 17.3)	0.22	7 (4)
Vol. contusions on MRI	236 (52)	1.36 (1.19; 1.55)	**< 0.001**	140 (80)	1.33 (1.13; 1.57)	**0.001**	50 (68)	1.63 (1.12; 2.37)	0.011	39 (71)	2.34 (1.21; 4.51)	0.011	7 (4)

All volume variables were in cm^3^ and transformed to their natural logarithms and for these analyses also a presence variable was included (since the natural logarithm of zero does not exist). Significant values, *p* <  0.01, are in bold

*GOSE* Glasgow Outcome Scale Extended, *TAI* traumatic axonal injury, *TBI* traumatic brain injury, *GCS* Glasgow Coma Scale, *n* numbers, *OR* odds ratio, *CI* confidence interval, *Vol.* volume, *No*. numbers, *FLAIR* fluid-attenuated inversion recovery, *DWI* diffusion-weighted imaging, T2*GRE/SWI T2* gradient echo or susceptibility-weighted imaging, *NA* not applicable (indicated if *n* < 10)

^a^ All analyses were adjusted for age and all analyses involving TAI variables for worst Marshall CT score

^b^ Ordinal logistic regression analyses with inverted 6 months GOSE score as the response variable for severe TBI and moderate TBI with GCS score 9–12. Binary logistic regression predicting disability (GOSE score ≤ 6) at 6 months for moderate TBI with GCS score 13. For all TBI, ordinal logistic regression analyses with inverted 6 months (moderate-severe TBI) or 3 months (mild TBI) GOSE scores

^c^ Due to low *n* (*n* < 10) for all MRI variables in the mild group, statistical analyses were not performed

^d^ Can also include patients with bilateral TAI in the brainstem and/or thalami

### Prognostic value of location of TAI and contusions

**Fig. 2:**
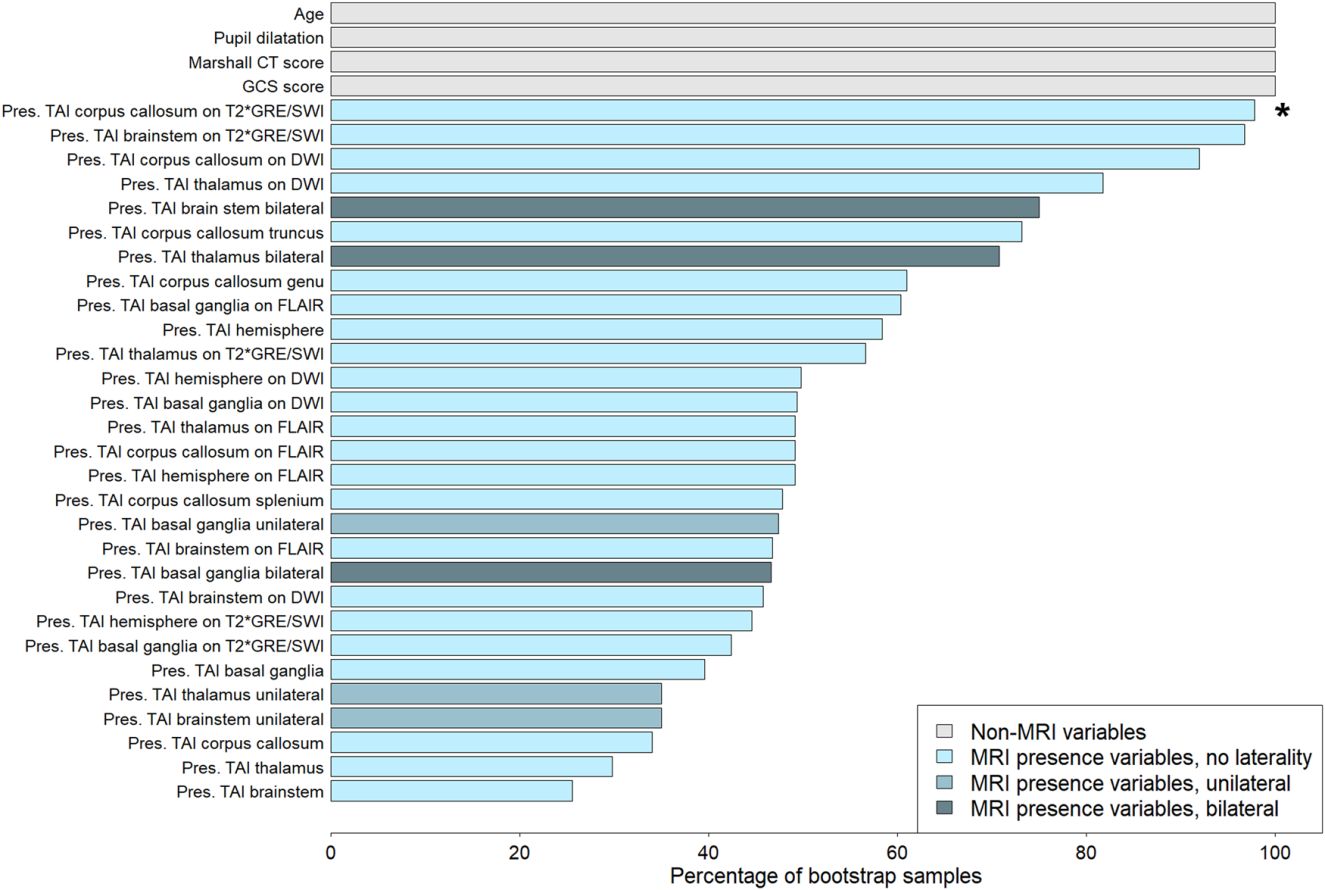
The elastic-net clinical TAI-MRI model where an ordinal regression model with elastic-net penalty is fitted to predict 6 months GOSE score in moderate-severe TBI. The model included TAI-MRI presence variables (including laterality variables). The worst Marshall CT score is always included in the model. The histogram shows the percentage of the 500 bootstrap samples for which each variable was included in the model (i.e. their coefficient was not set to zero). The plot is related to Supplemental Table S2. GCS, Glasgow Coma Scale; Pres., presence; TAI, traumatic axonal injury; T2*GRE, T2* gradient echo; SWI, susceptibility-weighted imaging; DWI, diffusion-weighted imaging; FLAIR, fluid-attenuated inversion recovery. * One variable (marked with *) had OR < 1 in elastic-net regression models (correlation phenomenon). Results for individual variables must be interpreted with caution since the joint effect of all variables together must be taken into consideration when interpreting this figure

In all TBI and severe TBI, bilateral TAI in thalami or brainstem in general and pons in particular (OR 10.7–11.7), were the location variables most strongly associated with worse outcomes according to the estimated ORs (Table 2). 75% with severe TBI and bilateral TAI in pons had poor outcomes (GOSE score ≤ 4). TAI unilaterally in the brainstem, thalamus, or basal ganglia had lower ORs with higher *p*-values than bilateral injuries (Table 2 and Supplemental Table S1). Bilateral TAI in the brainstem and thalami were included in > 70% of the bootstrap samples in the *elastic-net clinical TAI-MRI model* in msTBI, whereas bilateral TAI in basal ganglia and unilateral TAI in basal ganglia, brainstem, and/or thalamus were included in < 50% of the bootstrap samples (Fig. 2 and Supplemental Table S2). In all and severe TBI, TAI in the corpus callosum was also associated with outcome (Table 2).

In moderate TBI, none of the TAI presence variables were significantly associated with outcome (Table 2). In moderate TBI (including patients with GCS score 13), the presence of contusions on CT (OR 3.24 (95% CI 1.634; 6.43), *p* = 0.001) and volume of contusions on MRI (OR 1.65 (95% CI 1.24; 2.18), *p* = 0.001) significantly predicted GOSE score.

In mTBI, the presence of TAI did not significantly predict disability (GOSE score ≤ 6, OR 8.2 (95% CI 1.5; 44.6), *p* = 0.014, Supplemental Table S3). The presence of contusions and extra-axial haematomas predicted disability (*p* < 0.001). The wide CIs indicate large uncertainty in the estimated ORs in these analyses.

### Prognostic value of TAI numbers and volumes

**Fig. 3:**
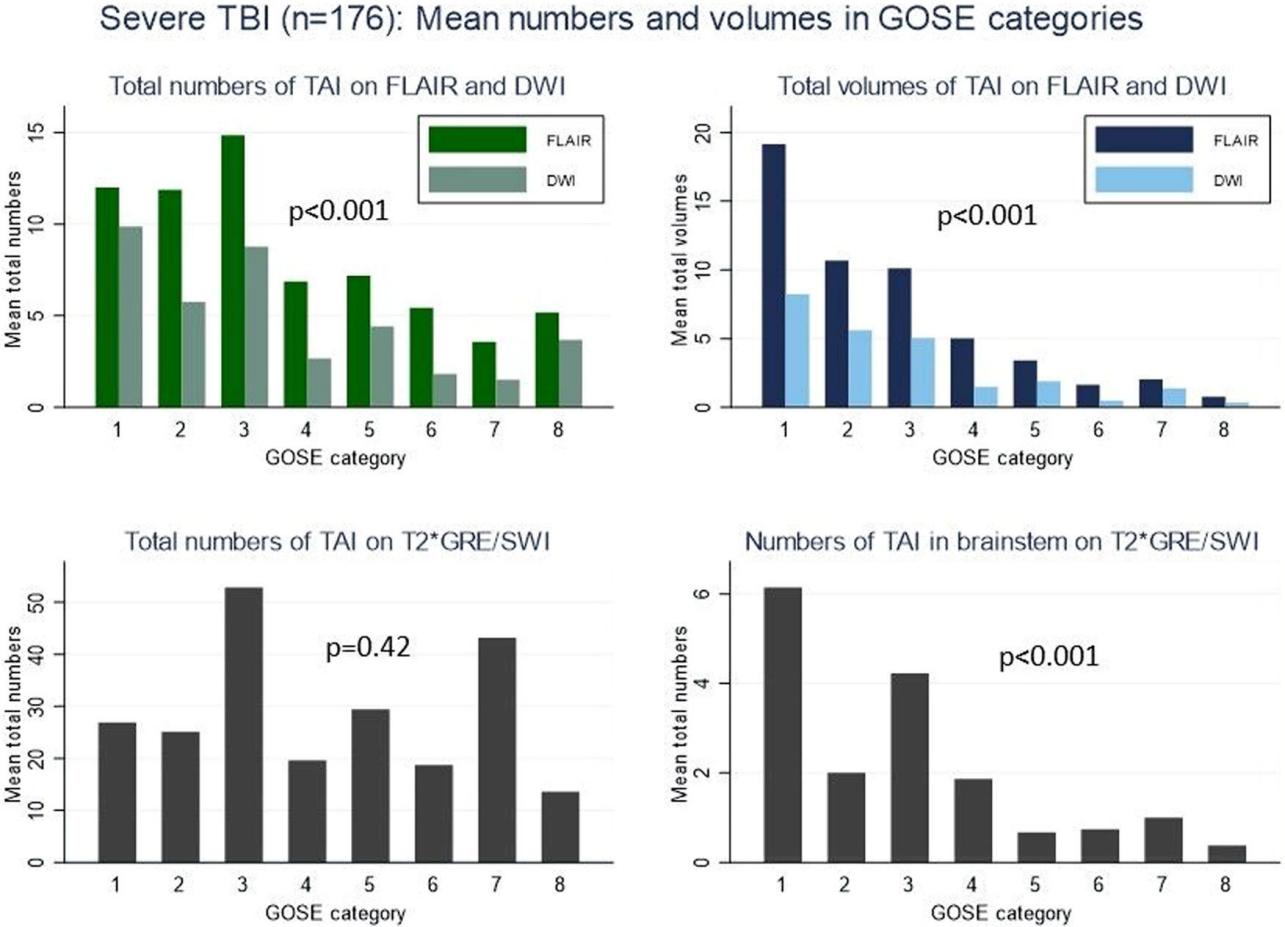
Mean numbers and mean volumes of TAI lesions in the different MRI sequences in severe TBI (*n* = 176). Top left: Mean total numbers of TAI on FLAIR (dark green) and DWI (light green). Top right: Mean total volumes of TAI on FLAIR (dark blue) and DWI (light blue). Bottom left: Mean total numbers of TAI on T2*GRE/SWI. Bottom right: Mean numbers of TAI in brainstem on T2*GRE/SWI (Only 8 patients with GOSE score 2). *p*-value indicates a trend for decreasing median (Jonckheere–Terpstra). TBI, traumatic brain injury; GOSE, Glasgow Outcome Scale Extended; TAI, traumatic axonal injury; FLAIR, fluid-attenuated inversion recovery; DWI, diffusion-weighted imaging; T2*GRE, T2* gradient echo; SWI, susceptibility-weighted imaging

In severe TBI, we found a negative association between total volumes and numbers of TAI on FLAIR and DWI, and GOSE score (Fig. 3). The same was found for the numbers of TAI in the brainstem on T2*GRE/SWI. In adjusted regression models in msTBI, TAI volumes explained the variance in outcome better than models with numbers (Table 3), where the model including total TAI volume on FLAIR (M7, Table 3) performed best. Also, in the *elastic-net quantitative TAI-MRI model* in msTBI, the total volume of TAI on FLAIR was included in 97% of the bootstrap samples (Supplemental Fig. 1 and Supplemental Table S4). Adjusting for the time between injury and MRI in the models including DWI, did not improve the model fit. The presence of TAI was low in several sublocations of the brain (Supplemental Table S5), leading to low statistical power.

**Table 3:** Moderate-severe TBI: ordinal logistic regression models (*n* = 305) with 6 months GOSE score as response variable and TAI number and volume variables on MRI as explanatory variables

M. No	Explanatory variables included in the model	No. of est. param.	Pseudo *R*^2^	AIC	BIC
M1	Core (age, pupil abnormalities, and GCS score)^a^	10	0.16	958	995
M2	Core^a^ + CT^b^	13	0.20	924	972
M3	Core^a^ + CT^b^ + No. TAI on T2*GRE/SWI	14	0.20	923	975
M4	Core^a^ + CT^b^ + No. TAI on DWI	14	0.21	918	970
M5	Core^a^ + CT^b^ + No. TAI on FLAIR	14	0.21	910	962
M6A	Core^a^ + CT^b^ + Vol. TAI on DWI	15	0.21	909	965
M6B	Core^a^ + CT^b^ + Vol. TAI on DWI + Interaction TAI on DWI and days to MRI^c^	17	0.22	912	975
**M7**	**Core**^**a**^ **+** **CT**^**b**^ **+** **Vol. TAI on FLAIR**	**15**	**0.22**	**900**	**956**
M8	Core^a^ + CT^b^ + Vol. TAI on FLAIR + Vol. TAI on DWI	17	0.23	901	964
M9	Core^a^ + CT^b^ + No. TAI on T2*GRE/SWI + Vol. TAI on FLAIR + Vol. TAI on DWI	18	0.23	903	970

*TBI* traumatic brain injury, *n* numbers, *GOSE* Glasgow Outcome Scale Extended, *TAI* traumatic axonal injury, *M.No* model number, *No* number, *est.param*. estimated parameters, *pseudo R*^2^ McFaddens pseudo R^2^, *AIC* Akaike information criterion, *BIC* Bayesian information criterion, *GCS* Glasgow Coma Scale, T2*GRE T2* gradient echo, *SWI* susceptibility-weighted imaging, *DWI* diffusion-weighted imaging, *FLAIR* fluid-attenuated inversion recovery, *Vol.* volume

^a^ Core variables were in line with the IMPACT prognosis calculator in TBI, see also ‘Materials and methods’

^b^ CT indicates the worst Marshall CT score

^c^ Since TAI lesions on DWI may attenuate over time, we included an interaction variable between DWI lesions and the number of days to MRI. None of these interaction variables were significant factors (*p* = 0.35–0.44).

The jointly assessed preferred TAI model row is shown in bold

The numbers and volumes of TAI were not significantly associated with outcomes in mild or moderate TBI (Table 2).

### The Trondheim TAI-MRI grading and quantitative models

**Fig. 4:**
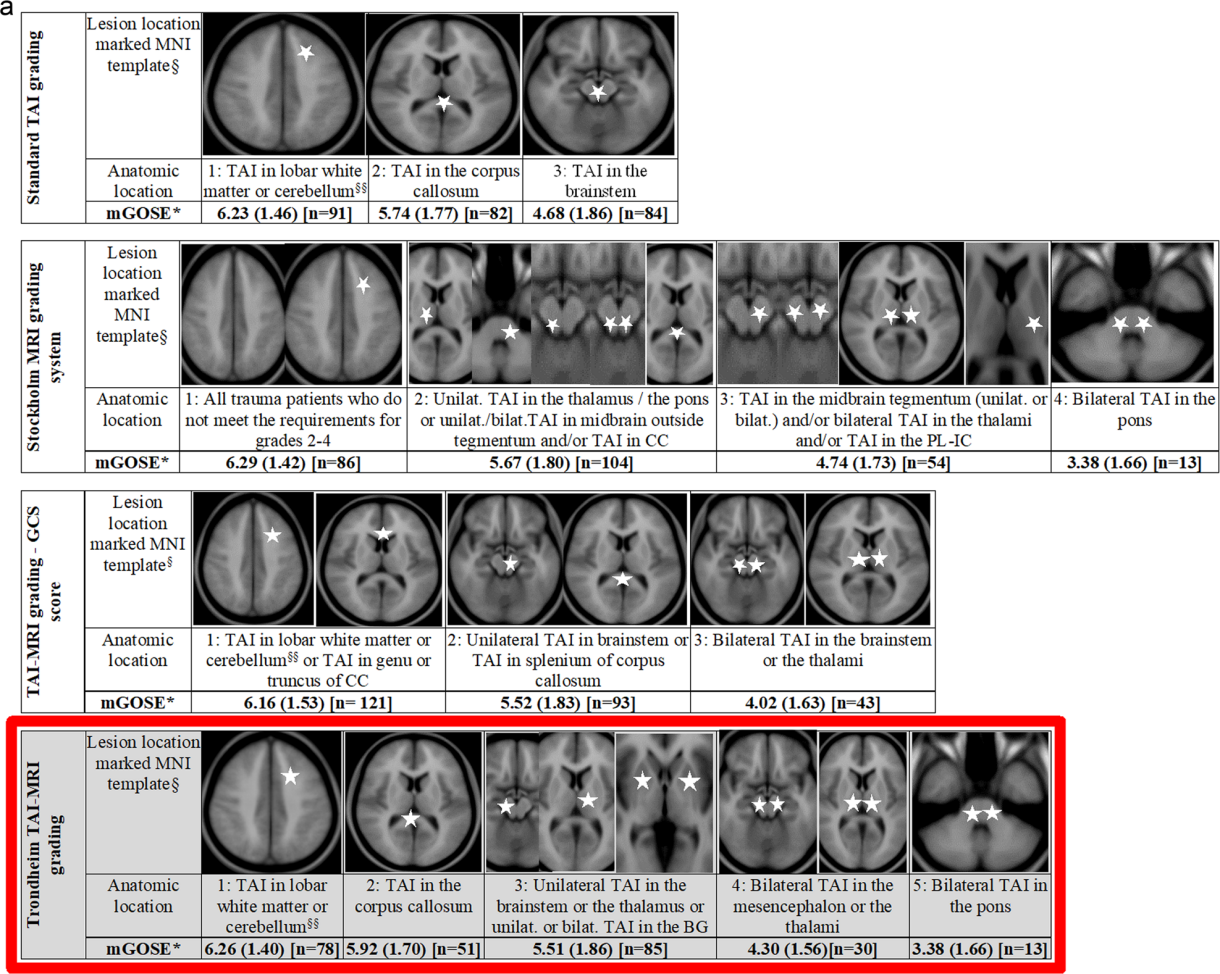

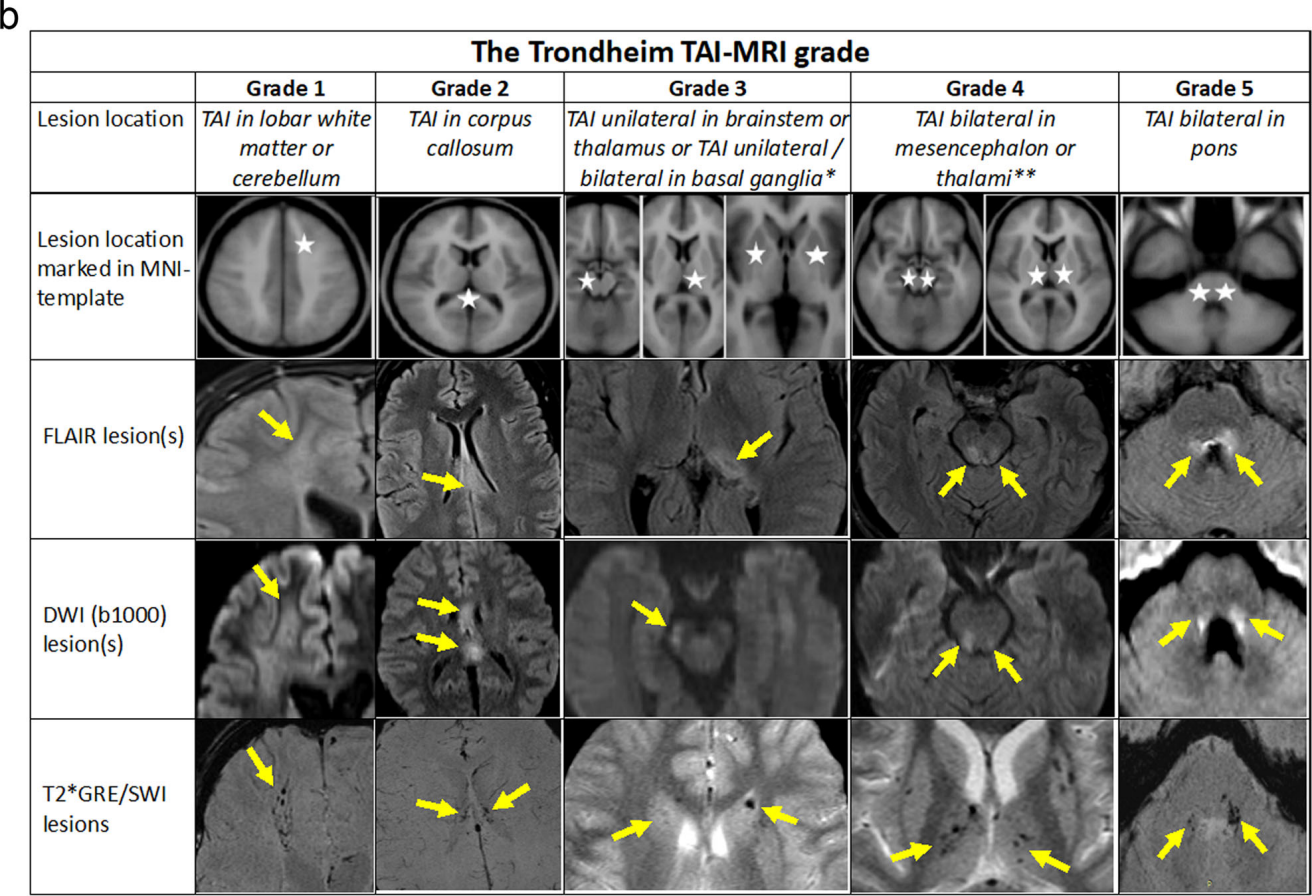
**a** Moderate-severe TBI#—Illustration of the proposed Trondheim TAI-MRI grading (marked with a red frame and light grey background colour) in relation to the other MRI gradings of TAI. The outcome indicated on the row below each grade, with mean GOSE* score at 6 months with (SD) and (numbers), shown for the different TAI gradings. #Mild TBI performed identically in all gradings, due to few TAI lesions. Only one patient had a lesion in the corpus callosum (GOSE score at 3 months of 8), and *n* = 8 had TAI grade 1 (mean GOSE 7.00 (SD 1.19)). § Permission to use, copy, modify, and distribute for any purpose if the above copyright notice appears—ICBM Copyright (C) 1993–2009 Louis Collins, McConnell Brain. §§ Since all patients in our material with TAI lesions in the cerebellum also had TAI lesions in the hemispheres, we have not illustrated the cerebellum in the different gradings. TBI, traumatic brain injury; TAI, traumatic axonal injury; mGOSE, mean Glasgow Outcome Scale Extended Score; *n*, numbers; unilat., unilateral; bilat., bilateral; CC, corpus callosum; PL-IC, posterior limb of the internal capsule; BG, basal ganglia. **b** Illustration of the Trondheim TAI-MRI grading with 5 grades. The lesion location is marked with white stars in an MNI-ICBM152 template in the 4th upper row. The rows below provide image examples of TAI lesions on FLAIR (5th row), DWI (b1000, 6th row), and T2*GRE/SWI (7th row). The patient’s lesion localisation with the highest TAI grade represents the final TAI grade. Note that lower-level locations of TAI may not necessarily be present. For TAI grades 1, 2, and 5 each column represents one patient. In grade 1, a patient with TAI in the right frontal white matter, visible in the three corresponding sequences. In grade 2, a patient with TAI in truncus of the corpus callosum in the three corresponding sequences. In grade 5, a patient with bilateral TAI in tegmental pons and the middle cerebellar peduncles is also visible in the three corresponding sequences. *For TAI grade 3, three different patients are shown to illustrate the different lesion localisations. In the 5th row, a FLAIR image from a patient with a unilateral TAI in the left pulvinar part of the left thalamus is shown. In the 6th row, a DWI (b1000) image from another patient with a unilateral TAI lesion in the left crus cerebri of mesencephalon is presented. In the 7th row, a T2*GRE image of a third patient with bilateral traumatic microbleeds in the caput of the caudate nuclei is displayed. **For TAI grade 4, two different patients are shown. The FLAIR and DWI images (rows 5 and 6) are from a patient with bilateral TAI in the tectum of the mesencephalon. In the 7th row, a T2*GRE image from another patient with bilateral traumatic microbleeds in the thalami as well as in the putamen (not indicated with arrows). TAI, traumatic axonal injury; FLAIR, fluid-attenuated inversion recovery; DWI, diffusion-weighted imaging; T2*GRE, T2* gradient echo; SWI, susceptibility-weighted imaging

Based on results from the preceding regression analyses, we propose the Trondheim TAI-MRI grading which is presented in Fig. 4a with three other TAI gradings on MRI [4, 12, 13]. In Fig. 4b, the Trondheim TAI-MRI grading is shown with image examples for each TAI grade and MRI sequence.

In severe TBI, pseudo *R*^2^ was higher (0.19 vs. 0.16) and AIC lower (571 vs. 581), when Trondheim TAI-MRI grading was compared to *standard* TAI grading (model A4 vs. A1, Table 4). Model fit was best in the quantitative model with a total volume of TAI and contusion on FLAIR (pseudo *R*^2^ 0.21, AIC 551) (TBI-FLAIR volume model, A6, Table 4).

**Table 4:** Different MRI gradings of TAI in various TBI injury severities: Adjusted^a^ ordinal and logistic regression analyses and cross-validated area under the curve (AUC)

				Model fit logistic regression model^b^			Cross-validated AUC^c^	
		Model	Variables included in the model	Pseudo *R*^2^	AIC	BIC	Mean AUC	Bootstrap 95% CI
Severe TBI	GCS score ≤ 8 *n* = 176	A0	Core variables and CT	0.15	585	626	0.80	0.68; 0.84
		A1	Standard TAI grading	0.16	581	632	0.81	0.73; 0.87
		A2	Stockholm MRI grading system	0.18	571	621	0.80	0.71; 0.86
		A3	TAI-MRI grading based on GCS score	0.17	577	627	0.81	0.71; 0.85
		A4	Trondheim TAI-MRI grading	0.19	571	628	0.84	0.72; 0.86
		*A5*	*Vol. TAI on FLAIR (TAI-FLAIR volume model)*	*0.20*	*555*	***602***	*0.86*	*0.76; 0.90*
		*A6*	*Vol. TAI on FLAIR+ Vol. Contusion on FLAIR (TBI-FLAIR volume model)*	***0.21***	***551***	*605*	***0.90***	***0.78; 0.91***
Moderate TBI	GCS score 9–12 *n* = 74	B0	Age, GCS score, and CT	0.15	214	**241**	0.76	0.69; 0.89
		B1	Standard TAI grading	0.16	219	253	0.69	0.64; 0.86
		B2	Stockholm MRI grading system	0.15	219	254	0.76	0.65; 0.86
		B3	TAI-MRI grading based on GCS score	0.16	219	253	0.73	0.63; 0.86
		B4	Trondheim TAI-MRI grading	0.17	219	258	0.77	0.63; 0.86
		B5	Vol. TAI on FLAIR (TAI-FLAIR volume model)	0.17	215	247	**0.78**	**0.64; 0.86**
		*B6*	*Vol. TAI on FLAIR + Vol. Contusion on FLAIR (TBI-FLAIR volume model)*	***0.19***	***213***	*250*	*0.76*	*0.61; 0.84*
	GCS score 13 *n* = 55	C0	Age and CT	0.15	67	73	0.66	0.51; 0.83
		C1	Standard TAI grading	0.15	69	77	0.72	0.50; 0.83
		C2	Stockholm MRI grading system	0.15	69	77	0.71	0.50; 0.83
		C3	TAI-MRI grading based on GCS score	0.16	69	77	0.76	0.47; 0.81
		C4	Trondheim TAI-MRI grading	0.15	69	77	0.73	0.51; 0.83
		C5	Vol. TAI on FLAIR (TAI-FLAIR volume model)	0.16	71	81	0.75	0.52; 0.85
		*C6*	*Vol. Contusion on FLAIR (Contusion-FLAIR volume model)*	***0.26***	***63***	***73***	***0.78***	***0.55; 0.90***
Mild TBI	GCS 14–15 *n* = 147	D0	Age and sex	0.05	102	111	NA	
		D4	TAI grading^d^ on MRI	0.09	100	112		
		D5	Vol. TAI on FLAIR (TAI-FLAIR quantitative model)	0.09	102	117		
		*D6*	*Vol. Contusion on FLAIR (Contusion-FLAIR volume model)*	***0.25***	***85***	***100***		

*TAI* traumatic axonal injury, *TBI* traumatic brain injury, *AUC* area under the curve, *GCS* Glasgow Coma Scale, *pseudo* *R*^2^ McFaddens pseudo *R*^2^, *AIC* Akaike information criterion, *BIC* Bayesian information criterion, *CI* confidence interval, *Vol.* volume, *FLAIR* fluid-attenuated inversion recovery, *NA* not applicable

^a^ Severe TBI is adjusted for core variables (age, pupil abnormalities and GCS score) and the worst Marshall CT score. Moderate TBI with GCS scores 9–12 are adjusted for age, GCS score and worst Marshall CT score. Moderate TBI with a GCS score 13 is adjusted for age and Marshall CT score. Mild TBI are adjusted for age and sex

For details on the contents and specifications for the different gradings, we refer to the Fig. 4

^b^ Ordinal logistic regression analyses with inverted 6 months Glasgow Outcome Scale Extended (GOSE) score as response variable for severe TBI and moderate TBI with GCS score 9–12. Binary logistic regression predicting disability (GOSE score ≤ 6) at 3 months for mild TBI and at 6 months for moderate TBI with GCS score 13

^c^ 10-fold cross-validation AUC analyses based on the logistic regression models but predicting poor outcome (GOSE score ≤ 4) for severe TBI and predicting disability (GOSE score ≤ 6) for moderate TBI. All values are given as mean AUC with 95% CI from the bootstrap samples

The most favourable model in each injury severity is indicated with italicised rows. The most favourable value in each category for each injury severity is in bold

^d^ Since patients with mild TBI only had TAI in the hemispheres or corpus callosum, all the clinical grading systems performed identically in this group

In moderate TBI (GCS score 9–12), none of the clinical TAI gradings were superior to the others, but pseudo *R*^2^ increased from 0.15 to 0.19 applying the TBI-FLAIR volume model (B6, Table 4). In patients with GCS scores 13–15, neither the presence nor volume of TAI improved model fit, while the Contusion-FLAIR volume model did (C6-D6, Table 4).

## Discussion

In this prospective study of all TBI severities with early MRI, we investigated the location, number, and volume of TAI lesions as potential predictors for outcome, after adjusting for established outcome predictors. In severe TBI, the presence of bilateral TAI in the brainstem or thalami was a strong outcome predictor, especially when located in pons. Interestingly, in mild-moderate TBI, the total volume of contusions on MRI was more important for outcome than TAI volume. Based on our results, we propose the Trondheim TAI-MRI grading (Fig. 4a, b) that can be applied by visual evaluation of early MRI. In all TBI severities, however, the best model fit was found when quantitative FLAIR models replaced the TAI-MRI grading.

We found that bilateral TAI in pons most strongly predicted worse outcomes at 6 months in severe TBI with ORs among the highest across the studied locations. In a retrospective MRI study of 255 critically ill TBI patients, the presence of bilateral TAI in pons was also proposed to represent the worst grade [12]. In a study from 2002, not specifically studying TAI, any bilateral MRI lesions in upper pons were the strongest predictor for mortality [29]. We therefore propose bilateral TAI in pons as the worst grade, Trondheim TAI-MRI grade 5.

The presence of bilateral TAI in mesencephalon or thalami was a strong outcome predictor in severe TBI. The thalamus consists mainly of grey matter nuclei but is surrounded by layers of WM and separated by a Y-shaped layer of WM, the internal medullary lamina [30], which may explain why TAI can be found in the thalamus. The thalamus is an important relay centre with reciprocal connections to nearly all parts of the brain, with the intralaminar nuclei embedded in the internal medullary lamina, particularly important for consciousness [30]. This can explain why bilateral TAI in the thalami is so important for the outcome. Also, we have previously found that patients with bilateral TAI in the thalami had lower GOSE scores than those with unilateral TAI in the thalamus [11], and bilateral TAI in the thalami was far more indicative of a low admission GCS than any other MRI finding [13]. In a DTI study, we found lower fractional anisotropy values in the thalamus in all standard TAI grades [31]. Also, two recent reviews on MRI in TBI concluded that any bilateral lesions in the brainstem or thalami increased the risk for poor outcomes [32, 33]. Finally, patients with bilateral TAI in the thalami were also associated with poor outcomes in the Stockholm MRI grading [12]. We propose that bilateral TAI in mesencephalon or thalami should be classified as Trondheim TAI-MRI grade 4.

Further, in all TBI analysed together, we found that unilateral TAI in the thalamus or brainstem and bilateral TAI lesions in the basal ganglia significantly predicted worse outcomes. It was expected that unilateral TAI in the brainstem or thalamus was not as important for the outcome as bilateral injuries, but we found it somewhat surprising that bilateral TAI in basal ganglia was not so closely associated with poor outcomes. However, the basal ganglia is primarily involved in motor control, while the brain stem and thalami are more important for vital functions and consciousness [11, 30]. We recently found that the presence of unilateral TAI in the brainstem was significantly associated with GCS score [13]. In moderate TBI with a GCS score of 9–12, the presence of any TAI in the brainstem, thalamus, or basal ganglia was not significantly associated with the outcome. However, the estimated OR (2.9) was similar to the one for severe TBI (OR 3.1), and the lower degree of evidence of an effect on outcome might be due to the lower frequency of TAI. Importantly, no patients with mTBI had TAI in the brainstem, thalamus, or basal ganglia. In a retrospective MRI study of 178 patients with severe TBI and TAI, multivariable ordinal regressions with adjustment for IMPACT variables also demonstrated the importance of any TAI in thalamus/basal ganglia for outcome at 12 months, in addition to TAI in the corpus callosum and brainstem [34]. We propose that the presence of unilateral TAI in the thalamus or brainstem or unilateral/bilateral TAI in basal ganglia should be classified as Trondheim TAI-MRI grade 3.

The presence of TAI in the corpus callosum was significantly associated with outcomes in severe TBI. However, many of these patients also had TAI in the brainstem, thalamus, or basal ganglia and it is difficult to deduct the contribution to outcome prediction. We did not find any evidence that TAI in the splenium was a stronger predictor of worse outcomes than TAI in genu/truncus, in contrast to the observed association with GCS score [13]. For clinical purposes, we therefore suggest that TAI in the corpus callosum is not further subdivided and is classified as grade 2. We also suggest that TAI in hemispheres or cerebellum still should be classified as grade 1, since there was little evidence in our data to recommend changing the current practice. Patients with mTBI almost exclusively only had TAI in the hemispheres; and in a larger sample, it is reasonable to anticipate that such lesions will be associated with outcomes even though we could not demonstrate a statistically significant effect.

In severe TBI, the Trondheim TAI-MRI grading performed better in predicting 6-month outcomes compared to the *standard* TAI grading, the Stockholm MRI grading [12] as well as our TAI-MRI grading based on GCS score [13]. The Stockholm MRI grading has a higher number of sublocations included in their grades 2 and 3, while the Trondheim TAI-MRI grading is more similar to the standard grading used today and thereby easier to learn and implement for the radiologist in everyday clinical practice. We also question that the Stockholm MRI grading does not distinguish patients without TAI on MRI from patients with TAI in hemispheres, since both will be allocated to grade 1 in that grading system.

In msTBI, the total volumes of TAI were more important outcome predictors than the total numbers, and volumes on FLAIR were more important than on DWI. Adjusting for the time factor on DWI did not improve model fit. We know from stroke imaging that DWI lesions disappear or attenuate 2–3 weeks after ictus [35], which is also the clinical experience in TBI. Thus, DWI is less useful in a clinical setting since MRI is typically performed later in msTBI than in stroke.

The prognostic model including TAI-FLAIR volumes gave high model fit in msTBI. The importance of TAI-FLAIR volumes in msTBI is supported by other smaller studies [10, 36, 37]. Interestingly, in all TBI severities, we found a better model fit generally when quantitative models replaced the clinical TAI-MRI grading. In moderate (GCS score 9-12) and severe TBI, the TBI-FLAIR volume model (including volume of TAI and contusion) gave the highest model fit, while in GCS score 13–15 the Contusion-FLAIR volume model contributed to the highest model fit. Smaller studies have earlier shown the predictive value of contusions in moderate [10] and mTBI [38]. The finding that FLAIR volumes gave even higher model fit than clinical MRI gradings, is promising for the use of artificial intelligence (AI) technologies. However, also in our models, a large proportion of the variance in the GOSE score remained unexplained. The outcome after TBI is multidimensional and assumed to be influenced not only by injury severity but also by other factors such as contextual factors and psychosocial functioning.

This study has several strengths: First, the prospective data collection and the large number of patients with early MRI. Second, we performed extensive structured template-based MRI readings and manual lesion segmentations on three different MRI sequences. Manual segmentation is regarded as the gold standard, automatic algorithms are promising but still not available for independent use [39]. Third, the MRI readings and segmentations were all performed blinded and quality-checked in inter-rater-analyses with good inter-rater-agreement [13].

One limitation is the selection bias that always will be present in early MRI studies of TBI, and we have earlier acknowledged reasons for this, such as age and injury severity [11, 14]. Even though the total sample is large, the lower number of patients together with the lower prevalence of MRI findings result in lower power in moderate and particularly mTBI. Another limitation is the heterogeneity of the MRI scanners with most patients examined with 1.5 T scanners in msTBI when preferably the whole cohort should have been imaged on 3 T. However, in a clinical setting, both 1.5 T and 3 T scanners will be used many years ahead and it is beneficial with a grading that can be used independently of field strength. Many of the msTBI patients in this cohort were examined with T2*GRE instead of SWI, which may have led to an underestimation of TAI. Thus, we recommend that the Trondheim TAI-MRI grading and the quantitative models will be externally validated in upcoming larger multicentre datasets with 3 T and SWI.

In conclusion, we propose the Trondheim TAI-MRI grading, with bilateral TAI in mesencephalon or thalami and bilateral TAI in pons as the worst grades 4 and 5, respectively. The Trondheim TAI-MRI grading most reliably estimated outcome in severe TBI, larger sample sizes will be necessary to clarify the importance in mild-moderate TBI. Interestingly, TAI seemed to be less important for outcome prediction in mild-moderate TBI where the volume of contusions on MRI predicted outcome better. The quantitative models comprising FLAIR lesion volumes, had the highest model fits in all TBI severities. In the future, the continuous improvements of AI will likely enable the use of quantitative models in the clinic. A more optimal prognostic classification of brain injury on early MRI will be important to help decision-making, informing patients and families, and stratifying patients for optimal follow-up.

## Supplementary information

ELECTRONIC SUPPLEMENTARY MATERIAL

## Abbreviations

AI: Artificial intelligence
AIC: Akaike information criterion
AUC: Area under the curve
BIC: Bayesian information criterion
CI: Confidence interval
CT: Computed tomography
DWI: Diffusion-weighted imaging
FLAIR: Fluid-attenuated inversion recovery
GCS: Glasgow Coma Scale
GOSE: Glasgow Outcome Scale Extended
MRI: Magnetic resonance imaging
NA: Not applicable
OR: Odds ratio
PL-IC: Posterior limb of internal capsule
SWI: Susceptibility-weighted imaging
T2*GRE: T2* gradient echo
TAI: Traumatic axonal injury
TBI: Traumatic brain injury
Vol: Volume
WM: White matter
