# Supplementary material for: The prognostic importance of traumatic axonal injury on early MRI: the Trondheim TAI-MRI grading and quantitative models
Source: Eur Radiol. 2024 Jun 19;34(12):8015–29. doi: 10.1007/s00330-024-10841-1 (PMC11557676; doi:10.1007/s00330-024-10841-1)
Supplement: Supplementary file 1 — ELECTRONIC SUPPLEMENTARY MATERIAL [file 330_2024_10841_MOESM1_ESM.pdf]

SUPPLEMENTAL MATERIAL

THE PROGNOSTIC IMPORTANCE OF TRAUMATIC AXONAL INJURY ON EARLY MRI: THE TRONDHEIM TAI-MRI GRADING AND QUANTITATIVE MODELS

SUPPLEMENTARY MATERIAL AND METHODS

This study consisted of 463 patients with TBI from three separate prospective cohorts (figure 1):

1) Trondheim mild TBI cohort, comprising patients (16-60 years) presenting with mild TBI to the emergency department at Trondheim University Hospital or at the Trondheim municipal outpatient clinic (April 2014-Descember 2015). Mild TBI was defined according to the WHO criteria[1]. Patients underwent MRI at median 2 (range 0-5) days.

2) Trondheim moderate-severe TBI cohort, comprising patients (8-70 years) admitted with either moderate (GCS score 9-13, or GCS score 14-15 and loss of consciousness > 5 min) or severe TBI (GCS score ≤ 8) according to the Head Injury Severity Scale (October 2004-October 2017)[2; 3]. Patients underwent MRI at median 9 (range 0-41) days.

3) Oslo severe TBI cohort comprising patients (16-70 years), admitted to Oslo University Hospital with severe TBI (GCS-score ≤ 8 during the first 24 hours post-injury) (January 2009-January 2011)[4]. Patients underwent MRI at median 5 (range 1-19) days.

Exclusion criteria

Exclusion criteria were: [1] age ≥ 70 years, to reduce the prevalence of age-related WMHs (white matter hyperintensities), and age < 8 years, due to difficulties assessing outcome according to Glasgow Outcome Scale Extended (GOSE) below this age; [2] MRI performed > 6 weeks post-injury, due to the attenuation of lesions on MRI over time[5]; [3] unreadable MRI due to large artefacts, one or more missing predefined MRI sequences (see below) or acute ischemia/infarction in large-vessel territories on diffusion MRI; and [4] inclusion in the CENTER-TBI project, since it will serve as a validation cohort in a planned follow-up multicentre study.

CT reading

The CT scans were reviewed by radiologists or residents in radiology in collaboration with neuroradiologists (Trondheim moderate-severe TBI cohort)[6], by reviewing the radiology report (Trondheim m-TBI cohort)[1], or by neuroradiologists (Oslo severe TBI cohort)[7]. In the Trondheim moderate-severe TBI cohort and Oslo severe TBI cohort, the worst CT scan was used and a Marshall CT score was assigned[8]. The worst CT scan was defined as the scan with the highest Marshall CT score with score 5 and 6 considered equally high. In the Trondheim mild TBI cohort all CT scans with intracranial traumatic pathology were reviewed once more by an experience neuroradiologist (KAK) and a Marshall CT score assigned. In the Trondheim mild TBI cohort, 31 patients were not found to be eligible for CT imaging in the acute phase based on the clinical evaluation[9] and they were assigned Marshall CT score of 1.

MRI acquisition and reading

The MRI protocols consisted of the following sequences: T1, T2, fluid attenuated inversion recovery (FLAIR), diffusion weighted imaging (DWI), and T2*gradient echo (T2*GRE, Trondheim moderate-severe TBI cohort: n=219 and Oslo severe TBI cohort: n=47) or susceptibility weighted imaging (SWI, Trondheim moderate-severe TBI cohort: n=43, Oslo severe TBI cohort: n=2, Trondheim mild TBI cohort: n=159).

MRI scans were scored and annotated blinded for clinical information by consultants in radiology (KGM, AMHF, EHS, ØO and SAB) according to the ERA-NET NEURON TAI-MRI template developed by the TAI-MRI partners. The presence and number of TAI lesions on FLAIR, DWI and T2*GRE/SWI were registered in 58 predefined locations in the brain[10].

Hypointense foci on T2*GRE/SWI were considered as signs of TAI, if they had the typical distribution, appearance, and location. Traumatic microbleeds often are asymmetric and occurs at predilection sites with an increasing gradient from hemispheres to deep brain [11], and thus show another distribution compared to for instance cerebral amyloid angiopathy [12] and hypertensive angiopathy [13]. Further, the appearance of traumatic microbleeds is typically linear, cigar, comet tail or bead like and they tend to appear in clusters [14; 15] in contrast to other cerebral microbleeds that are more scattered and round-ovoid [16]. Location of TAI is typical at the interface between grey and white matter, corpus callosum and brainstem [17; 18], but recently emphasized to be found in thalamus and basal ganglia as well [19; 20]. Bilateral

calcifications in basal ganglia are common but is often symmetric. In patients where SWI was available, the phase images were useful to distinguish calcifications from microbleeds.

Hyperintense foci on FLAIR and b1000 images on DWI were also considered as TAI lesions if they had a typical distribution, appearance, and location. Incidental white matter hyperintensities detected on the FLAIR as either periventricular hyperintensities (“caps and bands”) or patchy / confluent hyperintense lesions in deep white matter[21; 22], were considered unspecific and not trauma-related, and thus not included as TAI lesions. The brainstem might also be involved in chronic small vessel disease, but these lesions are usually diffuse, symmetrical, and central [23] while TAI is typically found in the posterolateral aspect of the brain stem [17].

In the evaluation and annotation of traumatic MRI findings in this study, the distribution, appearance, and location on all obtained MRI sequences were held together, in the same way that MRI sequences in the clinical radiological work-up of patients are considered complementary to each other. In those cases where the radiologist interpreted the lesions as non-traumatic and not as a sign of TAI, the lesion was not counted or segmented. If the radiologists were in doubt, they consulted one of the other radiologists to reach consensus.

If the lesion count exceeded 10 in one of the locations, the value 15 was assigned[5]. Patients were excluded if at least one of the essential MRI sequences (FLAIR, DWI and/or T2*GRE/SWI) were missing or unreadable[17]. The standard TAI grade was registered for all patients (see Introduction). Parenchymal changes following insertion of drain or intracranial pressure devices were defined as iatrogenic injuries, and thus not included to the TAI or contusion lesion number or volumes.

For a more comprehensive description on the MRI scanners, protocols and reading, we refer to a recent published related study[10].

Outcome and handling of missing outcome data

In mild TBI, outcome was assessed at 3-months by telephone or face-to-face with the Glasgow Outcome Scale Extended (GOSE), an instrument assessing global functioning after TBI on an eight-point scale.[24; 25] In moderate-severe TBI, outcome was assessed at 6-months with the GOSE, by telephone or face-to-face. In the Oslo severe TBI cohort, GOSE was administered

at 3 and 12 months, and 6-months GOSE score was calculated as a weighted mean, rounded to the nearest integer value: (2/3) x 3 months GOSE score + (1/3) x 12 months GOSE score.

In the moderate-severe TBI group, one patient had missing 12 months GOSE score, where the 3 months GOSE score was used. For three patients with missing 6 months GOSE score, the 12 months GOSE score was used. Sixteen patients (4.9%) were missing; not planned for follow-up (n=14), death of other reasons (n=1) or not possible to assess (n=1). We followed the recently published recommendations on how to handle missing outcome data in TBI [26-28]. We found no significant differences in baseline characteristics as age (p=0.47), sex (p=0.14), worst CT Marshall score (p=0.18) or GCS score (p=0.36) between the missing and the non-missing group. Little's Missing Completely at Random (MCAR) test showed that the MCAR assumption was not rejected (p=0.15), when including complete covariate data (age, pupil size, GCS score, CT Marshall score, secondary events and *standard* TAI grade in MRI). We imputed the missing values using a model-based approach (expectation-maximization algorithm), and the imputed values were rounded to the nearest integer values. Missing GOSE scores for 16 patients were imputed using this approach. A missing GCS score for one patient was imputed in a similar way. We had no reasons to assume the data was *missing not at random*. We anyway chose to perform sensitivity analyses by redoing some of the analyses with exclusion of the patients with missing GOSE scores (n=16). These analyses gave no changes to the results of the analyses.

For the mild TBI group, 11 patients (7%) had missing GOSE score at 3 months. Little's test indicated that the missing data was not MCAR (p=0.029) and with absent relevant auxiliary data, we chose to exclude these patients from the outcome analyses. None of those with TAI on MRI in the mild TBI group had missing GOSE scores at 3 months.

Statistical analyses

Since only 11 patients had bilateral pupil dilation, a collapsed binary categorical variable for pupil status (abnormal / normal) was used in the regression analyses. The Marshall CT score of the worst CT scan, was collapsed into four levels (1, 2, 3-4 or 5-6) in the regression analyses, due to low numbers in the categories 4 (n=4) and 6 (n=5). We found little evidence of an effect of centre (hospital) in preliminary analyses and therefore omitted centre from the multivariable analyses.

Based on assessment of model fit the volumes were log-transformed, while the numbers were used on the original scale. Since volumes of zero cannot be log-transformed, region specific binary variables indicating the presence or absence of TAI were also included in the regression models. Hence, an association between numbers or volumes and the GOSE score was estimated only for cases where TAI was present.

Penalized regression imposes a penalty to models with many predictors, where the aim is to avoid overfitting and reduce the complexity of the model. With these methods we can assess the *combined* value of the predictors rather than the predictive strength of each. Elastic-net regression, which is a combination of the limiting models ridge regression and lasso regression, typically performs well in the presence of highly correlated predictors[29]. To assess the ability of each of the elastic-net models to discriminate poor outcome (GOSE score ≤ 4) from favourable outcome (GOSE 5-8), we calculated an optimism-corrected area under the receiver operating characteristics (AUC-ROC)-curve as part of the bootstrap procedure described in the main document.

Data collection was performed in a web-based data collection system administered by the faculty of Medicine and Health Sciences at Norwegian University of Science and Technology (NTNU), Trondheim, Norway.

References

1 Einarsen CE, Moen KG, Haberg AK et al (2019) Patients with Mild Traumatic Brain Injury Recruited from Both Hospital and Primary Care Settings: A Controlled Longitudinal Magnetic Resonance Imaging Study. Journal of Neurotrauma. 10.1089/neu.2018.6360

2 Skandsen T, Kvistad KA, Solheim O, Strand IH, Folvik M, Vik A (2010) Prevalence and impact of diffuse axonal injury in patients with moderate and severe head injury: a cohort study of early magnetic resonance imaging findings and 1-year outcome. Journal of Neurosurgery 113:556-563

3 Stein SC, Spettell C (1995) The Head Injury Severity Scale (HISS): a practical classification of closed-head injury. Brain Injury 9:437-444

4 Andelic N, Anke A, Skandsen T et al (2012) Incidence of hospital-admitted severe traumatic brain injury and in-hospital fatality in Norway: a national cohort study. Neuroepidemiology 38:259-267

5 Moen KG, Skandsen T, Folvik M et al (2012) A longitudinal MRI study of traumatic axonal injury in patients with moderate and severe traumatic brain injury. Journal of Neurology, Neurosurgery and Psychiatry 83:1193-1200

6 Moe HK, Limandvik Myhr J, Moen KG, Haberg AK, Skandsen T, Vik A (2019) Association of cause of injury and traumatic axonal injury: a clinical MRI study of moderate and severe traumatic brain injury. Journal of Neurosurgery. 10.3171/2019.6.JNS191040:1-9

7 Roe C, Skandsen T, Anke A et al (2013) Severe traumatic brain injury in Norway: impact of age on outcome. J Rehabil Med 45:734-740

8 Marshall LF, Marshall SB, Klauber MR et al (1991) A new classification of head injury based on computerized tomography. Journal of Neurosurgery 75:S14-S20

9 Unden J, Ingebrigtsen T, Romner B, Scandinavian Neurotrauma C (2013) Scandinavian guidelines for initial management of minimal, mild and moderate head injuries in adults: an evidence and consensus-based update. BMC Med 11:50

10 Moe HK, Follestad T, Andelic N et al (2020) Traumatic axonal injury on clinical MRI: association with the Glasgow Coma Scale score at scene of injury or at admission and prolonged posttraumatic amnesia. Journal of Neurosurgery. 10.3171/2020.6.Jns20112:1-12

11 Meythaler JM, Peduzzi JD, Eleftheriou E, Novack TA (2001) Current concepts: diffuse axonal injury-associated traumatic brain injury. ArchPhysMedRehabil 82:1461-1471

12 Charidimou A, Boulouis G, Frosch MP et al (2022) The Boston criteria version 2.0 for cerebral amyloid angiopathy: a multicentre, retrospective, MRI-neuropathology diagnostic accuracy study. Lancet Neurol 21:714-725

13 Rubin A, Waszczuk Ł, Trybek G, Kapetanakis S, Bladowska J (2022) Application of susceptibility weighted imaging (SWI) in diagnostic imaging of brain pathologies - a practical approach. Clinical Neurology and Neurosurgery 221:107368

14 Iwamura A, Taoka T, Fukusumi A et al (2012) Diffuse vascular injury: convergent-type hemorrhage in the supratentorial white matter on susceptibility-weighted image in cases of severe traumatic brain damage. Neuroradiology 54:335-343

15 Rizk T, Turtzo LC, Cota M et al (2020) Traumatic microbleeds persist for up to five years following traumatic brain injury despite resolution of other acute findings on MRI. Brain Injury 34:773-781

16 Greenberg SM, Vernooij MW, Cordonnier C et al (2009) Cerebral microbleeds: a guide to detection and interpretation. Lancet Neurol 8:165-174

17 Gentry LR (1994) Imaging of closed head injury. Radiology 191:1-17

18 Parizel PM, Van Goethem JW, Ozsarlak O, Maes M, Phillips CD (2005) New developments in the neuroradiological diagnosis of craniocerebral trauma European Radiology 15:569-581

19 Moe HK, Moen KG, Skandsen T et al (2018) The Influence of Traumatic Axonal Injury in Thalamus and Brainstem on Level of Consciousness at Scene or Admission: A Clinical Magnetic Resonance Imaging Study. Journal of Neurotrauma. 10.1089/neu.2017.5252

20 Tjerkaski J, Nyström H, Raj R et al (2022) Extended Analysis of Axonal Injuries Detected Using Magnetic Resonance Imaging in Critically Ill Traumatic Brain Injury Patients. Journal of Neurotrauma 39:58-66

21 Matsusue E, Sugihara S, Fujii S, Ohama E, Kinoshita T, Ogawa T (2006) White matter changes in elderly people: MR-pathologic correlations. Magn Reson Med Sci 5:99-104

22 Griffanti L, Jenkinson M, Suri S et al (2018) Classification and characterization of periventricular and deep white matter hyperintensities on MRI: A study in older adults. Neuroimage 170:174-181

23 Kwa VI, Stam J, Blok LM, Verbeeten B, Jr. (1997) T2-weighted hyperintense MRI lesions in the pons in patients with atherosclerosis. Amsterdam Vascular Medicine Group. Stroke 28:1357-1360

24 Jennett B, Snoek J, Bond MR, Brooks N (1981) Disability after severe head injury: observations on the use of the Glasgow Outcome Scale. Journal of Neurology, Neurosurgery and Psychiatry 44:285-293

25 Wilson L, Boase K, Nelson LD et al (2021) A Manual for the Glasgow Outcome Scale-Extended Interview. Journal of Neurotrauma. 10.1089/neu.2020.7527

26 Richter S, Stevenson S, Newman T et al (2019) Handling of Missing Outcome Data in Traumatic Brain Injury Research: A Systematic Review. Journal of Neurotrauma 36:2743-2752

27 Gravesteijn BY, Sewalt CA, Venema E, Nieboer D, Steyerberg EW (2021) Missing Data in Prediction Research: A Five-Step Approach for Multiple Imputation, Illustrated in the CENTER-TBI Study. Journal of Neurotrauma. 10.1089/neu.2020.7218

28 Kunzmann K, Wernisch L, Richardson S et al (2020) Imputation of Ordinal Outcomes: A Comparison of Approaches in Traumatic Brain Injury. Journal of Neurotrauma. 10.1089/neu.2019.6858

29 Zou H, Hastie T (2005) Regularization and variable selection via the elastic net. Journal of the Royal Statistical Society Series B-Statistical Methodology 67:301-320

SUPPLEMENTAL TABLE S1 Moderate-severe TBI: Adjusted# ordinal logistic regression analyses (n=305) with 6 months GOSE score* as response variable and presence of TAI including laterality on clinical MRI as explanatory variables.

	Any MRI sequence			FLAIR		DWI		T2*GRE / SWI	
**Variable**	**n (%)**	**OR (95% CI)**	**p**	**OR (95% CI)**	**p**	**OR (95% CI)**	**p**	**OR (95% CI)**	**p**
TAI in hemispheres**	243 (80)	0.91 (0.52; 1.59)	0.732	1.15 (0.74; 1.80)	0.534	1.13 (0.74; 1.74)	0.571	1.00 (0.61; 1.65)	0.988
TAI in corpus callosum	142 (47)	1.17 (0.73; 1.87)	0.517	1.61 (1.00; 2.59)	0.050	1.97 (1.20; 3.23)	**0.007**	0.69 (0.42; 1.14)	0.148
Genu	40 (13)	1.32 (0.69; 2.50)	0.403	3.72 (1.74; 7.93)	**0.001**	4.74 (1.44; 15.5)	0.010	0.66 (0.28; 1.55)	0.336
Truncus	96 (31)	1.50 (0.94; 2.41)	0.090	1.90 (1.16; 3.12)	0.011	2.25 (1.30; 3.89)	**0.004**	0.88 (0.51; 1.51)	0.648
Splenium	97 (32)	1.23 (0.74; 2.03)	0.425	1.60 (0.96; 2.66)	0.069	2.14 (1.24; 3.69)	**0.006**	0.92 (0.47; 1.82)	0.821
TAI in brainstem									
Unilateral	52 (17)	1.38 (0.77; 2.48)	0.281	1.23 (0.64; 2.38)	0.540	0.87 (0.43; 1.73)	0.684	2.39 (1.17; 4.86)	0.017
Bilateral	32 (11)	3.24 (1.55; 6.81)	**0.002**	4.23 (1.74; 10.3)	**0.002**	5.13 (1.77; 14.9)	**0.003**	4.21 (1.75; 10.1)	**0.001**
TAI in pons									
Unilateral	23 (8)	1.64 (0.71; 3.78)	0.248	2.38 (0.87; 6.55)	0.093	1.85 (0.54; 6.30)	0.326	1.36 (0.44; 4.22)	0.598
Bilateral	13 (4)	6.30 (2.12; 18.7)	**0.001**	9.54 (2.61; 35.0)	**0.001**	NA		NA	
TAI in mesencephalon									
Unilateral	48 (16)	1.79 (0.99; 3.24)	0.054	1.21 (0.57; 2.58)	0.611	0.99 (0.47; 2.09)	0.974	3.37 (1.40; 8.11)	**0.007**
Bilateral	28 (9)	4.02 (1.82; 8.91)	**0.001**	3.76 (1.44; 9.81)	**0.007**	7.22 (2.07; 25.2)	**0.002**	6.90 (2.46; 19.4)	**<0.001**
TAI in thalamus									
Unilateral	42 (14)	1.57 (0.81; 3.05)	0.183	1.99 (0.90; 4.41)	0.089	3.86 (1.26; 11.86)	0.018	1.81 (0.88; 3.74)	0.107
Bilateral	19 (6)	4.29 (1.69; 10.9)	**0.002**	6.82 (2.01; 23.2)	**0.002**	10.23 (1.96; 53.4)	**0.006**	2.90 (0.95; 8.86)	0.061
TAI in basal ganglia									
Unilateral	43 (14)	1.71 (0.93; 3.13)	0.083	2.00 (0.99; 4.05)	0.055	1.65 (0.60; 4.54)	0.330	1.39 (0.75; 2.58)	0.298
Bilateral	16 (5)	2.22 (0.81; 6.14)	0.123	17.6 (2.15; 145.1)	**0.008**	4.89 (0.79; 30.4)	0.089	0.95 (0.28; 3.24)	0.929

TBI, traumatic brain injury; GOSE, Glasgow outcome scale extended; TAI, traumatic axonal injury; FLAIR, fluid-attenuated inversion recovery; DWI, diffusion weighted imaging; T2*GRE; T2* gradient echo; SWI, susceptibility weighted imaging; n, numbers; OR, odds ratio; CI, confidence interval; p, p-value; NA, not applicable.
# Adjustment for age, pupil abnormalities, GCS score and worst Marshall CT score.
* GOSE score at 6 months is dependent variable in multivariable ordinal logistic regression analyses. GOSE scores were inverted to give interpretable odds ratios. If 9 or fewer patients had lesions in a sub region, regression analyses were not performed (NA).
** Includes lesions in cerebellum, internal capsule, and external capsule.
Each column and row indicate one model. P-values <0.01 are in bold.

SUPPLEMENTAL TABLE S2 Moderate-severe TBI: Estimated odds ratios with 95% bootstrap confidence intervals, from elastic-net regression (α=0.8) predicting 6 months GOSE score. The results correspond to figure 2 the elastic-net clinical TAI-MRI model in the manuscript.

	**Variable**	**OR**	**95% CI**		**Percentage**
			**Lower**	**Upper**	**Not 0**
1	GCS score	0.78	0.70	0.84	100
2	*Marshall CT score 2 vs 1*	*2.88*	*1.42*	*7.32*	*100*
3	*Marshall CT score 3-4 vs 1*	*12.01*	*5.45*	*36.14*	*100*
4	*Marshall CT score 5-6 vs 1*	*5.89*	*2.56*	*17.4*	*100*
5	Pupil dilatation	3.03	1.42	7.19	100
6	Age	1.04	1.03	1.06	100
7	Pres. TAI corpus callosum on T2*GRE/SWI	0.59	0.27	1	98
8	Pres. TAI brainstem on T2*GRE/SWI	1.92	1	3.91	97
9	Pres. TAI corpus callosum on DWI	1.55	1	2.68	92
10	Pres. TAI thalamus on DWI	1.84	1	6.84	82
11	Pres. TAI brain stem bilateral	1.37	1	3.2	75
12	Pres. TAI corpus callosum truncus	1.17	1	2.52	73
13	Pres. TAI thalamus bilateral	1.51	0.88	3.36	71
14	Pres. TAI corpus callosum genu	1.01	0.77	2.27	61
15	Pres. TAI basal ganglia on FLAIR	1.18	0.72	2.42	60
16	Pres. TAI hemisphere	1	0.45	1.11	58
17	Pres. TAI thalamus on T2*GRE/SWI	1	0.66	2.4	57
18	Pres. TAI hemisphere on DWI	1	0.68	1.32	50
19	Pres. TAI basal ganglia on DWI	1	0.37	2.21	49
20	Pres. TAI hemisphere on FLAIR	1	0.67	1.47	49
21	Pres. TAI corpus callosum on FLAIR	1	0.8	1.96	49
22	Pres. TAI thalamus on FLAIR	1	0.52	2.22	49
23	Pres. TAI corpus callosum splenium	1	0.88	1.69	48
24	Pres. TAI basal ganglia unilateral	1	1	1.97	47
25	Pres. TAI brainstem on FLAIR	1	0.74	1.92	47
26	Pres. TAI basal ganglia bilateral	1	0.38	2.36	47
27	Pres. TAI brainstem on DWI	1	0.49	1.34	46
28	Pres. TAI hemisphere on T2*GRE/SWI	1	0.68	1.66	45
29	Pres. TAI basal ganglia on T2*GRE/SWI	1	0.45	1	42
30	Pres. TAI basal ganglia	1	1	1.85	40
31	Pres. TAI brainstem unilateral	1	0.56	1.09	35
32	Pres. TAI thalamus unilateral	1	0.55	1.22	35
33	Pres. TAI corpus callosum	1	0.43	1.01	34
34	Pres. TAI thalamus	1	0.82	1.63	30
35	Pres. TAI brainstem	1	0.95	1.43	26

TBI, traumatic brain injury; GOSE, Glasgow outcome scale extended; TAI, traumatic axonal injury; GCS, Glasgow coma scale; OR, odds ratio; CI, confidence interval; T2*GRE, T2* gradient echo; SWI, susceptibility weighted imaging; DWI, diffusion weighted imaging; FLAIR, fluid-attenuated inversion recovery.
We forced CT Marshall score to be included into the model, indicated with italic fonts.
OR=1 indicate that the variable was shrunken to zero and not included into the model; this is also marked with faded font color. The optimism corrected area under the curve for predicting poor outcome (GOSE score ≤ 4) was 0.910 (95%CI 0.890; 0.922) for this model.

**SUPPLEMENTAL TABLE S3. Mild TBI: Uni- and multivariable logistic regression models with GOSE score at 3 months as response variables and MRI findings as explanatory variables.**						
			**Univariable analyses (n=147)**		**Multivariable§ analyses (n=147)**	
	**Variable**	**n (%)***	**OR (95% CI)**	**p value**	**OR (95% CI)**	**p value**
**GOSE***	Any trauma pathology on MRI	16 (10)	8.13 (2.41; 27.48)	**0.001**	19.82 (4.10; 95.79)	**<0.001**
	-TAI lesions	9 (6)	4.81 (1.07; 21.52)	0.040	8.23 (1.52; 44.55)	0.014
	-Contusions	7 (4)	14.22 (2.84; 71.12)	**0.001**	29.00 (4.60; 182.72)	**<0.001**
	-Extra axial hematoma	6 (4)	14.88 (2.27; 97.34)	**0.005**	27.92 (3.48; 223.86)	**<0.001**

TBI, traumatic brain injury; GOSE, Glasgow outcome scale extended; n, numbers; OR, odds ratio; CI, confidence interval; TAI, traumatic axonal injury

*GOSE scores were dichotomized into good recovery (GOSE score 7-8) or disability (GOSE score ≤ 6), models predict disability.

§Multivariable analyses are adjusted for age and sex.

P-values <0.01 are in bold.

**SUPPLEMENTAL TABLE S4 Moderate-severe TBI: Estimated odds ratios with 95% bootstrap confidence intervals, from elastic-net regression (α=1.0) predicting 6 months GOSE score. The results correspond to supplemental figure 1 *the elastic-net quantitative TAI-MRI model* below.**					
	**Variable**	**OR**	**95% CI**		**Percentage not 0**
			**Lower**	**Upper**	
1	GCS score	0.79	0.72	0.86	100
2	*Marshall CT score 2 vs 1*	*2.23*	*1.06*	*5.96*	*100*
3	*Marshall CT score 3-4 vs 1*	*9.69*	*4.77*	*37.51*	*100*
4	*Marshall CT score 5-6 vs 1*	*5.45*	*2.55*	*19.16*	*100*
5	Pupil dilatation	2.99	1.75	8.39	100
6	Age	1.04	1.03	1.06	100
7	Pres. TAI corpus callosum on T2*GRE/SWI	0.59	0.25	0.88	99
8	Vol. TAI corpus callosum on DWI	1.33	1	2.25	97
9	Vol. TAI total on FLAIR	1.33	1	1.6	97
10	No. TAI basal ganglia on FLAIR	1.4	1	2.49	92
11	Vol. TAI thalamus on DWI	0.77	0.15	1	90
12	Pres. TAI brainstem on T2*GRE/SWI	1.66	1	2.81	89
13	Vol. TAI brainstem on DWI	1.19	1	2.56	85
14	Pres. TAI corpus callosum on DWI	1.17	1	2.76	81
15	Vol. TAI basal ganglia on DWI	1.11	1	3.1	76
16	No. TAI thalamus on T2*GRE/SWI	1.01	0.99	1.11	73
17	No. TAI hemisphere on DWI	1	0.9	1	73
18	No. TAI brainstem on FLAIR	1.07	1	1.28	72
19	Vol. TAI basal ganglia on FLAIR	1.03	1	1.96	70
20	Pres. TAI brainstem	1	1	2.19	64
21	No. TAI brainstem on DWI	1	0.97	1.41	63
22	Pres. TAI thalamus on T2*GRE/SWI	1	0.79	2.29	63
23	Vol. TAI total on DWI	1	0.91	1.34	62
24	No. TAI hemisphere on T2*GRE/SWI	1	0.99	1	62
25	Vol. TAI hemisphere on FLAIR	1	1	1.35	59
26	Pres. TAI corpus callosum genu	1	0.68	1.94	59
27	No. TAI total on FLAIR	1	1	1.03	59
28	Vol. TAI corpus callosum on FLAIR	1	0.86	1.3	58
29	Vol. TAI thalamus on FLAIR	1	0.87	1.85	57
30	Pres. TAI basal ganglia unilateral	1	1	2.18	56
31	Pres. TAI hemisphere	1	0.45	1.26	55
32	Pres. TAI hemisphere on FLAIR	1	0.77	1.7	55
33	No. TAI corpus callosum on FLAIR	1.03	0.96	1.14	53
34	Pres. TAI hemisphere on T2*GRE/SWI	1	0.75	1.95	53
35	Pres. TAI corpus callosum on FLAIR	1	1	1.97	52
36	Vol. TAI brainstem on FLAIR	1	0.83	1.47	52
37	No. TAI thalamus on DWI	1	0.5	1	51
38	Pres. TAI corpus callosum truncus	1	0.83	1.65	51
39	No. TAI basal ganglia on T2*GRE/SWI	1	0.96	1.05	49
40	Pres. TAI hemisphere on DWI	1	0.61	1.33	49
41	Pres. TAI brain stem bilateral	1	0.9	2.31	48
42	Pres. TAI corpus callosum splenium	1	0.58	1.18	47
43	Vol. TAI hemisphere on DWI	1	0.8	1.17	47

44	Pres. TAI thalamus bilateral	1	0.36	1.82	45
45	Pres. TAI brainstem unilateral	1	0.96	1.82	44
46	Pres. TAI basal ganglia	1	1	1.82	44
47	Pres. TAI basal ganglia bilateral	1	0.39	1.84	43
48	Pres. TAI thalamus unilateral	1	0.69	1.65	42
49	Pres. TAI thalamus on FLAIR	1	0.4	1.09	42
50	No. TAI brainstem on T2*GRE/SWI	1	0.96	1.04	42
51	No. TAI corpus callosum on T2*GRE/SWI	1	0.98	1.01	41
52	No. TAI hemisphere on FLAIR	1	0.99	1.03	41
53	No. TAI basal ganglia on DWI	1	0.78	1.57	38
54	Pres. TAI basal ganglia on T2*GRE/SWI	1	0.52	1.11	36
55	No. TAI corpus callosum on DWI	1	0.91	1.1	35
56	Pres. TAI basal ganglia on DWI	1	0.62	1.96	33
57	Pres. TAI corpus callosum	1	0.65	1.41	32
58	Pres. TAI brainstem on DWI	1	0.57	1.4	32
59	Pres. TAI brainstem on FLAIR	1	0.63	1.51	31
60	Pres. TAI thalamus on DWI	1	0.67	2.2	31
61	No. TAI thalamus on FLAIR	1	0.8	1.11	31
62	No. TAI total on DWI	1	0.96	1	30
63	Pres. TAI thalamus	1	0.74	1.35	27
64	No. TAI total on T2*GRE/SWI	1	1	1	24
65	Pres. TAI basal ganglia on FLAIR	1	0.71	1.29	21

TBI, traumatic brain injury; GOSE, Glasgow outcome scale extended; GCS, Glasgow coma scale; OR, odds ratio; CI, confidence interval; TAI, traumatic axonal injury; T2*GRE, T2* gradient echo; SWI, susceptibility weighted imaging; DWI, diffusion weighted imaging; FLAIR, fluid-attenuated inversion recovery.
We forced CT Marshall score to be included into the model, indicated with italic fonts.
OR=1 indicate that the variable was shrunken to zero and not included into the model; this is also marked with faded font color. The optimism corrected area under the curve for predicting poor outcome (GOSE score ≤ 4) was 0.925 (95%CI 0.908; 0.938) for this model.

SUPPLEMENTAL TABLE S5 Moderate-severe TBI: Adjusted# ordinal logistic regression analyses with 6 months GOSE score* as response variable and TAI lesion burden in different locations and sub locations on MRI as explanatory variables (n=305).

	**TAI lesions on FLAIR**			**TAI lesions on DWI**			**TAI lesions on T2*GRE / SWI**	
**Location**	**n (%)**	**Number OR (95% CI)**	**Volume OR (95% CI)**	**n (%)**	**Number OR (95% CI)**	**Volume OR (95% CI)**	**n (%)**	**Number OR (95% CI)**
**Hemispheres**	189 (62)	1.04 (1.00; 1.08)	**1.41 (1.16; 1.71)**	121 (40)	1.02 (0.95; 1.10)	1.24 (0.95; 1.61)	216 (71)	1.00 (0.99; 1.02)
ICEC	13 (4)	3.09 (0.36; 26.5)	0.93 (0.45; 1.91)	7 (2)	NA	NA	20 (7)	1.16 (0.97; 1.40)
Cerebellum**	17 (6)	1.01 (0.53; 1.91)	0.93 (0.55; 1.56)	14 (5)	**5.36 (1.52; 18.9)**	2.60 (0.80; 8.44)	30 (10)	0.96 (0.84; 1.10)
**Corpus Callosum**	123 (40)	**1.24 (1.08; 1.43)**	**1.67 (1.30; 2.15)**	93 (31)	**1.23 (1.03; 1.48)**	**1.85 (1.36; 2.52)**	88 (29)	**1.04 (1.01; 1.06)**
Genu	24 (8)	0.51 (0.21; 1.23)		10 (3)	0.32 (0.08; 1.25)		23 (8)	**1.14 (1.01; 1.28)**
Truncus	81 (27)	1.18 (0.87; 1.61)		56 (18)	1.32 (0.76; 2.30)		61 (20)	1.03 (0.99; 1.07)
Splenium	86 (28)	**1.57 (1.07; 2.31)**		71 (23)	1.32 (0.77; 2.68)		39 (13)	**1.13 (1.01; 1.26)**
Fornix	15 (5)	2.47 (0.66; 9.22)	0.72 (0.25; 2.10)	2 (1)	NA		7 (2)	NA
**Brainstem**	60 (20)	**1.50 (1.21; 1.86)**	**2.07 (1.40; 3.05)**	47 (15)	**1.64 (1.16; 2.32)**	**2.90 (1.66; 5.06)**	54 (18)	1.04 (0.99; 1.09)
Mesencephalon	44 (14)	**1.73 (1.13; 2.65)**	**1.77 (1.11; 2.82)**	36 (12)	1.36 (0.95; 1.95)	**2.27 (1.14; 4.54)**	36 (12)	1.05 (0.95; 1.17)
Crus Cerebri	24 (8)	1.55 (0.30;7.97)		19 (6)	1.63 (0.83; 3.22)		26 (9)	0.98 (0.81; 1.20)
Tegm/SN	35 (12)	1.70 (0.58; 4.98)		23 (8)	2.01 (0.57; 7.05)		25 (8)	1.14 (0.90; 1.44)
Tectum	16 (5)	0.65 (0.11; 3.97)		7 (2)	NA		14 (5)	1.15 (0.97; 1.36)
Pons	25 (8)	**2.18 (1.22; 3.91)**	1.67 (0.98; 2.85)	14 (5)	**6.57 (1.79; 24.1)**	**3.53 (1.42; 8.81)**	18 (6)	1.05 (0.91; 1.21)
Basilar pons	12 (4)	0.38 (0.04; 2.05)		8 (3)	NA		1 (0)	NA
Tegmentum	16 (5)	3.99 (0.97; 16.4)		7 (2)	NA		16 (5)	0.98 (0.70; 1.36)
Cer. ped	11 (4)	**22.2 (2.07; 237.4)**		8 (3)	NA		7 (2)	NA
Med. Oblongata	1 (0)	NA		0	NA		1 (0)	NA
**Thalamus**	39 (13)	1.76 (1.00; 3.11)	**1.70 (1.03; 2.79)**	19 (6)	0.69 (0.22; 2.17)	1.26 (0.60; 2.63)	46 (15)	**1.05 (1.00; 1.10)**
Anterior	9 (3)	NA		5 (2)	NA		12 (4)	1.01 (0.85; 1.21)
Lateral	25 (8)	2.90 (0.44; 19.0)		13 (4)	4.14 (0.37; 45.7)		22 (7)	1.14 (0.95; 1.38)
Medial	19 (6)	**4.84 (1.90; 12.4)**		5 (2)	NA		24 (8)	**1.20 (1.02; 1.41)**
Pulvinar	14 (5)	**3.74 (1.27; 11.1)**		6 (2)	NA		15 (5)	**1.17 (1.05; 1.30)**
**Basal ganglia**	33 (11)	**8.56 (2.33; 31.5)**	**2.08 (1.15; 3.74)**	19 (6)	2.43 (0.65; 9.03)	**2.49 (1.08; 5.78)**	50 (16)	0.99 (0.93; 1.05)
N.caudatus	6 (2)	NA		7 (2)	NA		11 (4)	0.44 (0.14; 1.43)
Putamen	20 (7)	10.2 (0.53; 199.0)		9 (3)	NA		30 (10)	1.12 (0.98; 1.28)
Globus Pallidus	10 (3)	2.47 (0.45; 13.5)		5 (2)	NA		19 (6)	0.92 (0.84; 1.01)
Claustrum	3 (1)	NA		2 (1)	NA		6 (2)	NA
**TOTAL**	221 (72)	**1.07 (1.03; 1.10)**	**1.61 (1.34; 1.92)**	172 (56)	**1.07 (1.02; 1.12)**	**1.61 (1.29; 2.02)**	231 (76)	1.01 (1.00; 1.01)

TBI, traumatic brain injury; GOSE, Glasgow outcome scale extended; TAI, traumatic axonal injury; FLAIR, fluid-attenuated inversion recovery; DWI, diffusion weighted imaging; T2*GRE, T2* gradient echo; SWI, susceptibility weighted imaging; n, numbers; OR, odds ratio; CI, confidence interval; ICEC, internal and external capsule; Tegm/SN, tegmentum and substantia nigra, Cer.ped, cerebellar peduncles; N.caudatus; nucleus caudatus; NA, not applicable.
# Adjustment for age, pupil abnormalities, Glasgow coma scale score and worst Marshall CT score.
* GOSE score at 6 months is dependent variable in multivariable ordinal logistic regression analyses. GOSE scores were inverted to give interpretable odds ratios. If 9 or fewer patients had lesions in a sub region, regression analyses were not performed (NA).
**All patients with TAI in the cerebellum, also had TAI elsewhere in the cerebral hemispheres.
Each column and row indicate one model. Bold text indicates that the 95% CI does not contain zero. *

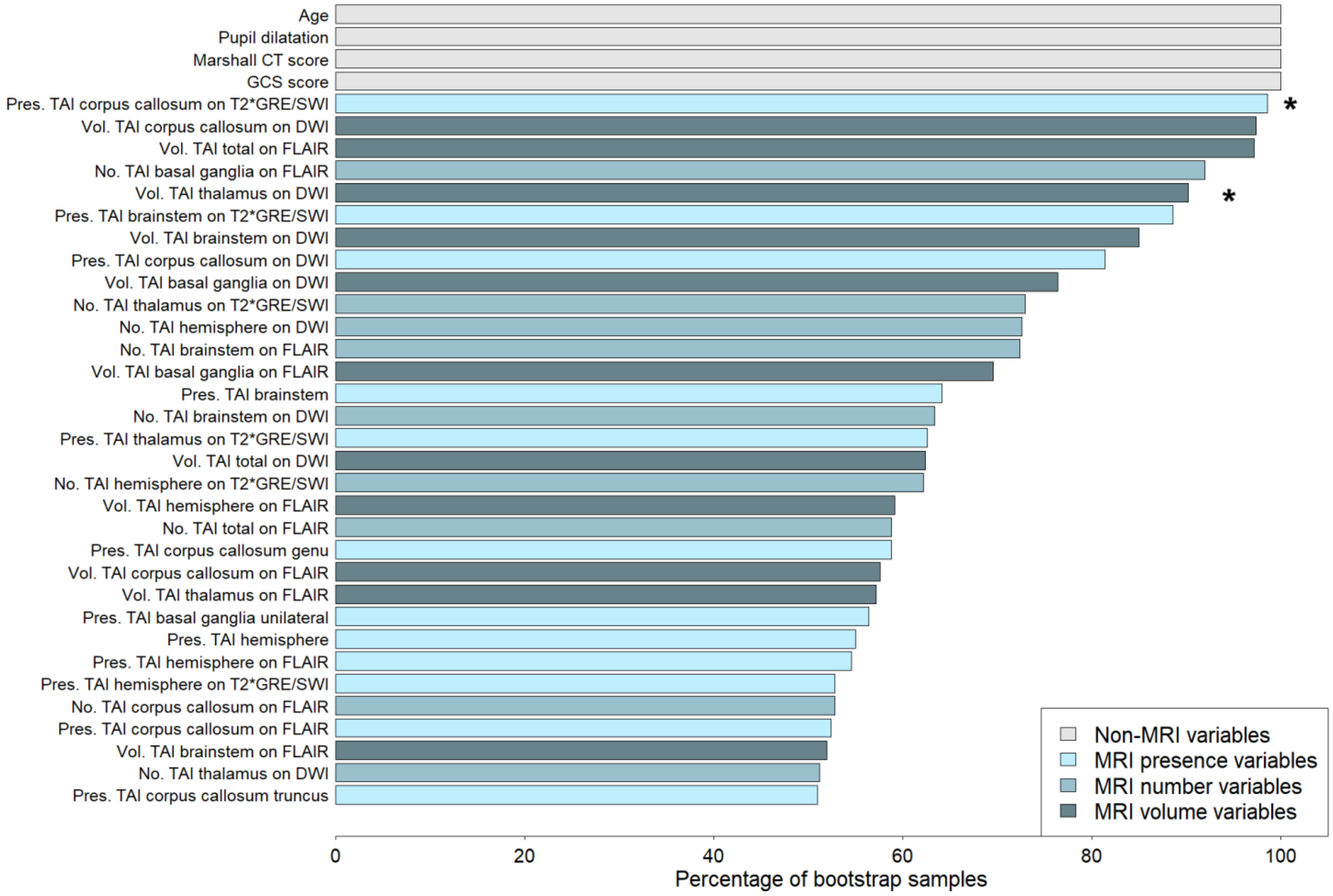

SUPPLEMENTAL FIGURE 1. The *elastic-net quantitative TAI-MRI model.* An ordinal regression model with elastic-net penalty was fitted to predict 6 months GOSE score in moderate-severe TBI. The model included TAI-MRI presence variables (including laterality variables) *and* TAI-MRI number and volume variables. Worst Marshall CT score are always included into the model. The histogram shows the percentage of the 500 bootstrap samples for which each variable was included in the model (i.e. their coefficient was not set to zero). For simplicity, only variables selected in at least 50% of the bootstrap samples are shown. The plot is related to supplemental table S4. In figure 2 (main document) and supplemental table S2, the *elastic-net clinical TAI-MRI model* is presented.

GCS, Glasgow Coma Scale; Pres., presence; Vol., volume; No., number; TAI, traumatic axonal injury; T2*GRE, T2* gradient echo; SWI, susceptibility weighted imaging; DWI, diffusion weighted imaging; FLAIR, fluid-attenuated inversion recovery.

* Two variables (marked with *) had OR<1 in elastic-net regression models (correlation phenomenon). Results for individual variables must be interpreted with caution since the joint effect of all variables together must be taken into consideration when interpreting this figure.
